# Analytical Methods for Determination of Phytic Acid and Other Inositol Phosphates: A Review

**DOI:** 10.3390/molecules26010174

**Published:** 2020-12-31

**Authors:** Gregor Marolt, Mitja Kolar

**Affiliations:** Faculty of Chemistry and Chemical Technology, University of Ljubljana, Večna pot 113, SI-1000 Ljubljana, Slovenia; gregor.marolt@fkkt.uni-lj.si

**Keywords:** phytic acid, inositol hexaphosphate, inositol phosphates, analytical methods, potentiometric titrations, ion-exchange chromatography, high performance liquid chromatography, spectroscopy, biosensors, nanosensors

## Abstract

From the early precipitation-based techniques, introduced more than a century ago, to the latest development of enzymatic bio- and nano-sensor applications, the analysis of phytic acid and/or other inositol phosphates has never been a straightforward analytical task. Due to the biomedical importance, such as antinutritional, antioxidant and anticancer effects, several types of methodologies were investigated over the years to develop a reliable determination of these intriguing analytes in many types of biological samples; from various foodstuffs to living cell organisms. The main aim of the present work was to critically overview the development of the most relevant analytical principles, separation and detection methods that have been applied in order to overcome the difficulties with specific chemical properties of inositol phosphates, their interferences, absence of characteristic signal (e.g., absorbance), and strong binding interactions with (multivalent) metals and other biological molecules present in the sample matrix. A systematical and chronological review of the applied methodology and the detection system is given, ranging from the very beginnings of the classical gravimetric and titrimetric analysis, through the potentiometric titrations, chromatographic and electrophoretic separation techniques, to the use of spectroscopic methods and of the recently reported fluorescence and voltammetric bio- and nano-sensors.

## 1. Introduction

**Figure 1:**
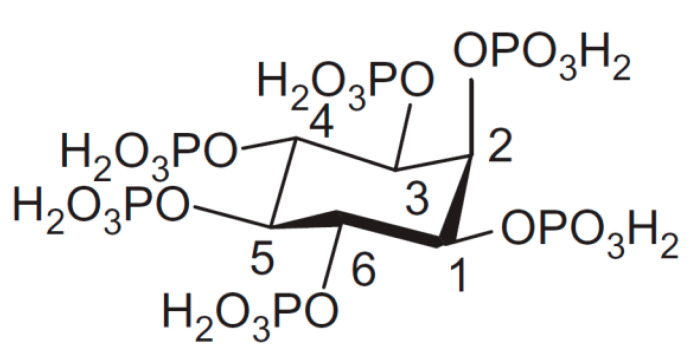
Structure of phytic acid in equatorial conformation.

Phytate (InsP6) represents a deprotonated (salt) form of dodecaprotic phytic acid (Figure 1) which can also be found in the literature by other names, including the commonly used inositol hexakisphosphate (generally abbreviated as InsP6, IP6), 1,2,3,4,5,6-hexakis(dihydrogenphosphate) *myo*-inositol, or by the following IUPAC name; (1s,2R,3R,4r,5S,6S)-cyclohexane-1,2,3,4,5,6-hexayl hexakis(dihydrogen (phosphate)). Chemically, it is a six-fold dihydrogenphosphate ester of *myo*-inositol or *cis*-1,2,3,5-*trans*-4,6-cyclohexanehexol which is the most abundant of nine possible isomers of inositol (Ins). *Myo*-orientation is also found in the case of phytic acid, which is due to the fact that the maximal number (i.e., five out of six) of phosphate groups are present in thermodynamically stabilized equatorial position [1]. However, the molecule can be inverted from equatorial (1a5e) to the axial (5a1e) orientation between pH 9.0 and pH 9.5 [2,3].

Because of the structural, chemical and physical properties, phytates can be found in many biological systems; including most of plant and mammalian cells [4]. In nature they exist mostly in the form of calcium–magnesium–potassium mixed salts, also known by the term phytins [5]. The highest content of phytates were found in plant seeds and grains (e.g., cereals, legumes, and nuts [6]) as the main source of inositol and phosphorous (typically accounting for 60–90% of total P) [7], as well as an important storage of cations (in the form of phytate salts) and high-energy phosphoryl groups [8]. Although the biological role of phytic acid in the animal cells has not been fully explained, it has been shown to serve several important physiological functions; including antioxidant activity [9], cell signalling [10], and regulation of different intracellular processes [11].

Particularly in the case of (vegetarian) diets based on the plant products, such as wheats and legumes that are very frequent in developing countries, phytates can play an important role in nutrition. Numerous publications regarding their antinutritional effects can be found in the literature due to their strong binding interactions with essential minerals, such as Ca^2+^, Mg^2+^, Zn^2+^, Fe^2+^, Fe^3+^, Cu^2+^, and Mn^2+^ [12], proteins, carbohydrates and lipids [13]. This is mostly due to the electrostatic interactions which arise from the (partial) deprotonation of phytic acid in the wide range of pH and consequent negative charge of phytate anion in the range from −1 to −12 [14]. Formation of (un)soluble coordination compounds decreases the bioavailability of minerals in food and hinders their absorption [15]. Phytates can also form unspecific complexes with some proteins and therefore change their solubility and enzymatic activity [16], inhibit carbohydrate metabolism [17], whereas the formation of lipophytic products leads to formation of metallic soaps and decreased lipid bioavailability [18]. Because humans and most of animals (except ruminants [19]) are not able to digest phytic acid, phytase is added into animal food [20], while specific microorganism, which can synthesize this enzyme, are used in the case of human nutrition [21]. Phytases catalyze the hydrolysis of phytic acid (InsP6) which is dephosphorylated to lower *myo*-inositol phosphates (represented as InsP*x*, *x* < 6), namely: *myo*-inositol pentakis- (InsP5), tetrakis- (InsP4), tris- (InsP3), bis- (InsP2), monophosphate (InsP1), and in certain cases to the final product *myo*-inositol (Ins), leading to weaker interaction with nutrients and decreased antinutritional activity [22]. However, non-enzymatic hydrolysis can also take place when food is heated (e.g., autoclaving, canning) or treated with strong acid [23]. However, phytic acid also functions as a precursor of inositol pyrophosphates (*x* > 6), such as InsP7 and InsP8, in which the fully phosphorylated InsP6 ring is further phosphorylated to create molecules that contain one or more high-energy pyrophosphate bonds [24].

On the other hand, strong interactions with heavy metals [1], particularly with iron and copper ions [14], exhibit also an antioxidant [25] and anticancer effects [26] of phytates alone or in combination with other inositol phosphates as reviewed by Vučenik and Shamsuddin [27] (2006). A specific coordination site between phosphate groups 1, 2, and 3 (see Figure 1) causes a significant negative shift of reversible redox potential of the redox couple Fe^2+^/Fe^3+^ [28] and even more importantly the removal of all available (six) coordination sites of iron, and therefore completely inhibiting its ability to catalyze Fenton reaction and hydroxyl radical (^•^OH) formation [29]. As a result, there are many studies of therapeutic or other beneficial effects of phytic acid as a dietary agent [27]. Moreover, phytates exhibit also a number of other beneficial effects on human health, such as inhibition of kidney stone formation [30] and reducing the risk of cardiovascular diseases by lowering of serum cholesterol level [31].

**Scheme 1:**
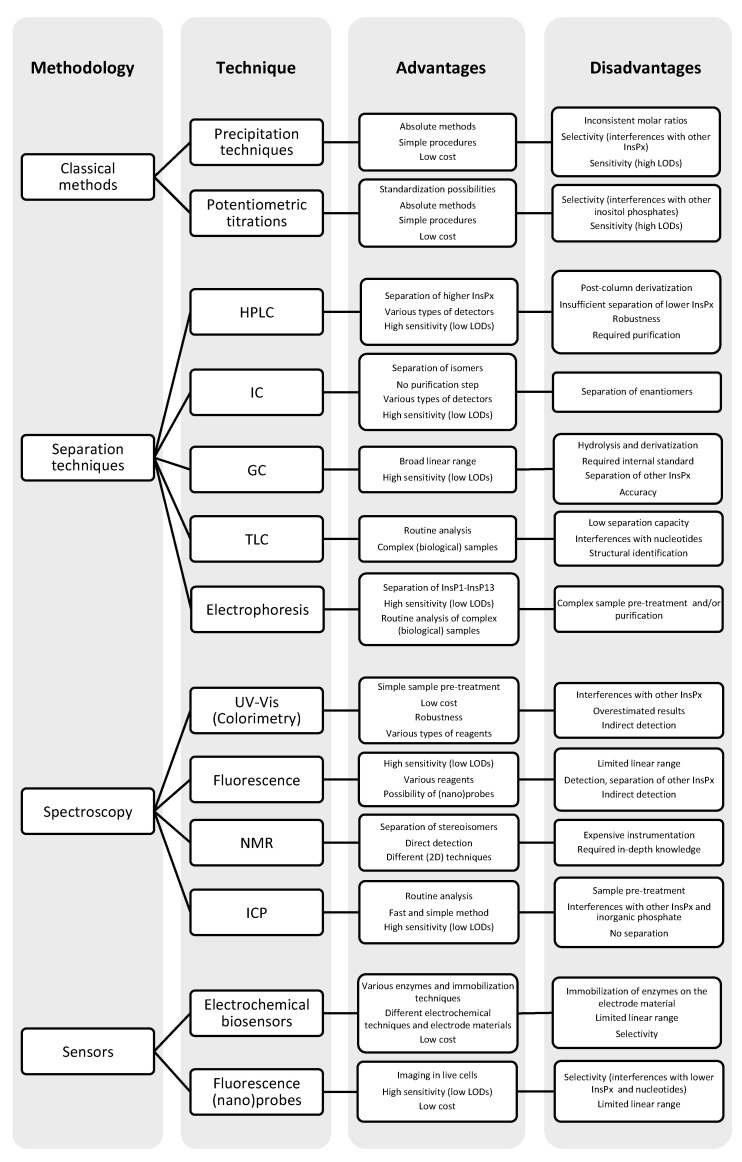
An overview of the most important analytical methodologies and techniques for the analysis of phytic acid and other inositol phosphates with the corresponding advantages and disadvantages of each method.

Diverse (biological) roles, applications [32], and biomedical utilization of inositol phosphates as well as their significance in various fields of research escalated the demands for the development of suitable analytical methods which would enable: (i) separation and (ii) quantification of these intriguing analytes in different types of the sample matrix. This review was prepared with the focus on the development of analytical procedures and methods that have been used for the isolation and determination of phytic acid and other inositol phosphates from the very beginning of the classical precipitation-based analysis, throughout the potentiometric titrations, separation and spectroscopic methods, to the recently reported bio- and nano-sensor applications. Where possible, different detection systems are discussed (particularly in the case of chromatography) and the limits of detection (LODs) are compared. For a better overview, the main analytical methodologies and techniques with corresponding advantages and limitations, reviewed and discussed later in this work, are summarized in Scheme 1.

## 2. Classical Analytical Methods

### 2.1. Precipitation Techniques

The first (original) analytical method for the determination of phytic acid content in cereals was developed in 1914 by Heubner and Stadler [33], which was based on the extraction of finely ground grain particles by HCl and titration of the extract with acidic solution of FeCl_3_ in the presence of ammonium thiocyanate. Phytate forms white precipitate with Fe^3+^ ions, while the titration endpoint is observed by the appearance of red-colored iron(III) thiocyanate complex. To overcome difficulties with the endpoint recognition, improvements of the original method were introduced in the following years by Rather [34] (1917), Averill and King [35] (1926), and Harris and Mosher [36] (1934) implementing modifications into direction of total phosphorous assay and/or quantification of precipitated iron. As an example of an early classical analysis of phytic acid from this period is worth mentioning the publication of McCance and Widdowson [37] (1935) who used the approach based on the extraction of dried food samples with HCl, filtration, and neutralization with NaOH to prepare the sample solution which was precipitated with FeCl_3_. The precipitate was then separated from the heterogenous mixture by filtration and upon the addition of NaOH and heating, the iron(III) was transferred from the initial phytate complex to Fe(OH)_3_ precipitate, while the released phytate solution was hydrolyzed by Kjeldahl wet digestion method [38] and used for the colorimetric determination of orthophosphate according to the procedure of Briggs [39] (1922) based on the molybdenum blue reaction [40]. The procedure was used for the assay of phytic phosphorous in different foodstuffs and its absorption in humans, but it was later replaced due to the tedious and time-consuming procedure.

A faster assay can be achieved by an indirect method, where phytate is again firstly precipitated with heating in the acidic solution of a known iron(III) content and secondly the excess of iron is determined by back titration. An additional improvement of the method, as originally described by Young [41] (1936), is colorimetrical determination of iron 2,2′-bipyridine complex at 519 nm in order to determine the decreased iron in the supernatant which is proportional to the phytic acid content. A similar approach was later used for the analysis of phytate in soya-based vegetable protein by Davies and Reid [42] (1979) and in cereal and cereal products by Haug and Lantzsch [43] (1983) with modifications of previously described procedure of Holt [44] (1955). As reported by Reeves et al. [45] (1979) thorium(IV) has also been used for the titrimetric assay of phytate as an additional reagent to the commonly used iron(III). As discussed in the following paragraphs, the use of the first chromatographic methods began in the same period, however the precipitation methods were still more convenient for the routine (food) analysis, although the chromatography showed better accuracy as discussed also by Thompson and Erdman [46] (1982) who investigated the comparison between both methods for determination of phytate in soybeans.

In general, precipitation reaction of phytic acid is not dependable and shows difficulties for analytical applications due to the following main reasons. (i) The first and most important issue is an inconsistent stoichiometric ratio between iron(III) and phytic acid in the precipitate, which depends strongly on the pH, ionic strength and the presence of other multivalent metals, such as Ca^2+^, exhibiting a synergistic effect on the amount of the precipitate formed [46]. (ii) The second problem is the non-selectivity as the titrimetric methods do not allow to distinguish between phytate and its dephosphorylated analogues (InsP5–InsP1) and/or inorganic (poly)phosphates which can also form insoluble precipitates with Fe^3+^ ions and thus cause overestimations. (iii) Finally, for these kind of (classical) titration procedures very large quantities of the dried biological material (e.g., plants or seeds) are required for reasonable titrant consumptions. Due to these fundamental problems of precipitation-based techniques, the results from this period should be considered carefully, particularly in the case of analysis of food samples which are rich in both the multivalent metals and phytate hydrolysis products, i.e., InsP5–InsP1 and “free” orthophosphate. Additional information regarding the development of early methods for phytic acid determination in foodstuffs can be also found in the review of Xu et al. [47] (1992).

### 2.2. Potentiometric Titrations

**Figure 2:**
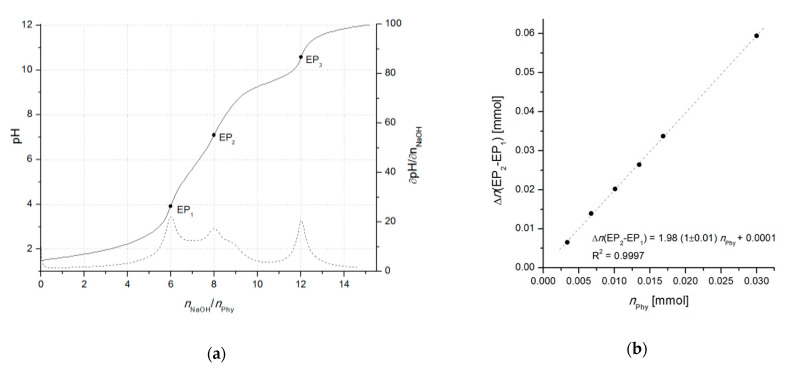
(**a**) Titration curve of 0.3081 mmol of phytic acid in 1.0 M NaCl with 0.0996 M NaOH, accompanied by the corresponding derivative ∂pH/∂*n*(NaOH). Equivalent points (three in total) are indicated as EP_1_, EP_2_, and EP_3_, and correspond to the 6th, 8th, and 12th deprotonation step, respectively. (**b**) Calibration curve based on the linear dependance of the difference between the titrant consumption at first (EP_1_) and second (EP_2_) equivalent point (∆n(EP_2_–EP_1_)) on the number of moles of titrated phytic acid (*n*_Phy_). The differential method allows for a reliable standardization of phytic acid regardless on the initial protonation level of phytate. The calculation of phytate amount can be performed also by using other pairs of equivalent points, e.g., EP_1_–EP_3_ or EP_2_–EP_3_. Adopted from [50].

With the development of advanced computational data analysis (around year 2000), titrimetric methods have been extensively applied also for the investigation of phytate acid-base properties as well as its interactions with multivalent metal ions, resulting in numerous publications of protonation and complex stability constants, collected in the reviews of Torres et al. [12] (2005) and Crea [48] et al. (2007). Due to the uncertainty of phytic acid concentration, which can arise because of the insufficient purity of commercially available phytate salts (< 95%) and/or fast moisture sorption, the use of accurate analytical methods requires a particular attention to the precise standardization of phytic acid prior to its use as a standard and/or for the calibration of the method. This can be achieved by potentiometric titration using an one-point determination as described by Luján and Tong [49] (2015). However, due to the problematic determination of the initial phytate protonation level which can lead to higher experimental errors, the differential (two-point) alkalimetric determination, introduced by Marolt and Pihlar [50] (2015), proved as a reliable standardization procedure of phytic acid (Figure 2). The method was later applied in the study of the phytate complexation with monovalent and divalent metals by Marolt et al. [14] (2020) and for the investigation of the removal of dissolved organic phosphorous (in the form of phytate) from wastewaters by Petzoldt et al. [51] (2020).

## 3. Chromatography

### 3.1. Liquid Chromatography (LC)

Separation methods were developed in order to overcome the issues with precipitation-based determination of phytate, that can be due to environmental factors and (thermal) pretreatment [52,53] leading to phytate hydrolysis and formation of lower inositol phosphates which also precipitate with iron(III) and therefore lead to false indication and/or overestimated phytate content. First attempt of paper chromatography separation of inositol phosphates was reported in 1956 by Desjobert and Petek [54] (1956) who used the isolation method by Posternak and Posternak [55] (1929) and claimed to separate seven hydrolysate products of phytate. In the same period, a chromatographic method using a column containing an ion-exchange resin was first introduced by Smith and Clark [56] (1952) and applied for separation of nine enzymatically produced derivatives of phytic acid by stepwise elution with HCl solution. The method was modified by Cosgrove [57] (1963) to separate also the InsP5 with the use of a strong anion exchange column (Dowex 1). After the perchloric acid digestion, the analysis of soil samples based on the determination of molar ratio between inositol and phosphorous, determined by the biological and colorimetrical assay, respectively. In 1976 Isaacks et al. [58] further improved the method for determination of phosphorylated metabolic intermediates (including InsP5) in chicken blood samples using the UV-detection at 260 nm and wet ash phosphate determination according to the work by Bartlett [59] (1959).

In the early 80s, the development of different separation techniques brought also the first applications of high-performance liquid chromatography (HPLC) for the analysis of food samples, as reported by Tangendjaja et al. [60] (1980) who determined phytic acid content in rice bran. As reported also by food studies of Camire and Clydesdale [61], Knuckles et al. [62], and Graf and Dintzis [63] (all in 1982) the separation was performed on the reversed-phase columns and a differential refractive index (RI) detector was used for the analysis of different inositol phosphates. However, the applicability of these methods was limited due to the difficulties with separation of inositol phosphates and quantification as the solvent front coincided with the phytic acid peak. This issue was improved markedly by Lee and Abendroth [64] (1983), who introduced the ion-pair concept by the addition of tetrabutylammonium hydroxide into mobile phase, and furtherly applied by Sandberg and Ahderinne [65] (1986) demonstrating that InsP3–InsP6 could be separated by adjusting the pH from 4.3 to 7.1. Burbano et al. [66] (1994) developed a methodology for simultaneous determination of phytic acid and higher inositol phosphates (InsP5–InsP3) content in the most important types of legumes in the Mediterranean diet using the additional purification step with strong anion-exchange column for the removal of lower inositol phosphates (InsP1 and InsP2) and analysis by ion-pair chromatography on C18 reversed-phase column. Optimal protonation levels of analytes were achieved by moderate acidity of the mobile phase (pH 4.3) as previously reported by Lehrfeld [67] (1989). It was shown that the use of ion-pair reagents, higher pH values, and lower percentage of methanol in mobile phase increases the retention times on the reverse phase columns and allows for the separation of InsP3–InsP6. Detection with refractive index showed identical RFs for both commercially available standards (InsP3 and InsP6) which were used also for the quantification of other inositol phosphates assuming the same detector sensitivity. However, the reported linear ranges were rather narrow (1–2 orders of magnitude) with high LOD (> 0.1 mg/mL).

Findings about new biological functions of inositol triphosphates (InsP3) as a second messenger in cellular signal transduction [68] in 1984 and consequently increased needs for efficient separation of also lower inositol phosphates resulted in numerous publications [69,70,71,72] of HPLC anion-exchange chromatographic methods for separation of InsP1–InsP3 and other organic (poly)phosphates. However, due to the isotopic labeling and fluorescence detection, these methods were mostly applied for biological investigations and rarely for the food analysis. In 2008 Letcher [73] et al. applied HPLC separation after enzymatical conversion of InsP6 to InsP7 (or more precisely to [PP]InsP5) and ^32^P-labeling which increased the sensitivity of radioactivity detection with the LOD down to 250 fmol equaling to 5 nM of InsP6 in urine. The method was applied for the phytic acid assay in various types of biological samples, including HeLa cells, rat tissues, slime mold, human white cells, serum, plasma and urine. Due to the use of recombinant InsP6 kinase, the method is highly selective for the analysis of phytic acid and does not allow detection of lower inositol phosphates. The most widely used protocol to study the metabolism of inositol (poly)phosphates is [^3^H]-inositol labeling coupled with chromatographic separation, as originally reported by Azevedo and Saiardi [74] (2006). The procedure consists of incubation of yeast cells with tritiated inositol which is taken up and metabolized into different phosphorylated forms, followed by an acidic extraction of soluble (poly)phosphates, strong anion exchange high-performance liquid chromatography (SAX-HPLC) separation, and radioactive detection using manual scintillation counting of individual fractions. With the use of minor modifications, this routine protocol has been applied for the determination of InsP1–InsP8 also in other types of biological samples, including mammalian cells and plant seedling [75].

### 3.2. Ion-Exchange Chromatography (IC)

Despite fairly satisfactory results of HPLC analysis of inositol phosphates, demands for less complicated and more specific methods with satisfactory robustness arose due to the well-known phytic acid dependence on the sample matrix, especially selected metal ions and proteins. Moreover, relatively sophisticated instrumentation is required for the HPLC methods which was (at the time) not widely accessible in many analytical laboratories. Therefore, in 1985 Phillippy and Johnston [76] (1985) applied the ion chromatography for determination of phytic acid in different foodstuffs by the use of SAX column (AS3) and inline detection by iron(III) complexation which reveals the absorption peak at 290 nm and allows for low detection limit of phytic acid (around 1 nmol). To prevent the iron phytate precipitation, which is favorable at higher pH and lower phytate-to-iron molar ratios [50], the use of HNO_3_ as an eluent is required in order to provide an acidic and non-complexing medium for Fe^3+^ ions (log *K* = −0.22) [77].

In 1994 Frühbeck et al. [78] omitted the ion-exchange chromatographic purification step with the modification of a rapid indirect spectrometric method of Vaintraub et al. [79] (1988), which was based on the colorimetric analysis of unpurified extracts of plant seeds, and in combination with the colorimetric method of Lattta and Eskin [80] (1980) developed a precise and reproducible methodology for extraction and determination of phytic acid in legumes and other food products. The method was comprised of a standard 2–3 h extraction of the ground sample material (< 0.7 mm) with HCl solution at pH 0.6 in order to release the phytate from its iron and protein complexes according to the reports of Sandberg et al. [81] (1993). After centrifugation the samples were adjusted to pH 6.0 with NaOH, which is above the protein isoelectric point, and run through an anion-exchange column (AG 1X4). Inorganic phosphate (i.e., orthophosphate) was eluted by 0.1 M NaCl, and a 7-fold higher concentration of NaCl was used for elution of phytate. The detection was conducted with the modified Wade reagent [82], which is the mixture (or more precisely a complex) of FeCl_3_ and sulfosalicylic acid and exhibits an absorption maximum around 500 nm [80]. In the presence of phytic acid, iron(III) is converted to the more stable phytate complex, resulting in a decreased absorbance of initial sulfosalicylic acid complex, which gave the linear response of the spectrophotometer in the concentration range of 5–50 µg/mL and limit of detection around 0.02% of phytate. A similar detection principle has been also used before by Cilliers and Niekerk [83] (1986) and Rounds and Nielsen [84] (1993), who developed a post-column reaction method for inline detection of phytate and other inositol phosphates. However, the peak resolution of lower inositol phosphates (InsP1–InsP3) and orthophosphate was unsatisfactory.

Extraction procedures of inositol phosphates require well controlled experimental conditions, particularly the pH, as it can be used to reduce the disturbing influence of metal ions and proteins in the sample matrix. In certain cases, addition of other ligands, such as ethylenediaminetetraacetic acid (EDTA) in the work of Bos et al. [85] (1991), was evaluated during the extraction but did not give the satisfactory results as its metal complexation requires alkaline pH because of relatively high p*K*_a_ values of final two deprotonation steps of EDTA (p*K*_a5_ = 6.13; p*K*_a6_ = 10.37) [86].

Development of first non-radiometric methods, that allowed separation of different inositol phosphate stereoisomers, took place in the mid-1980s with the publication of Meek [87] (1986) who coupled an anion-exchange chromatographic system (Pharmacia Mono Q HR 5/5 column) to a post-column reactor loaded with immobilized alkaline phosphatase. The online enzymatic hydrolysis and subsequent reaction with molybdate reagent was used for the analysis of InsP2–InsP4 by monitoring the phosphate absorption peek at 830 nm. Using a strong anion-exchange Mono-Q column and gradient elution with hydrochloric acid, Mayr [88] (1988) developed an isomer-selective method allowing for separation of total 20 inositol phosphates (retention time < 90 min), and much higher sensitivity by a so-called metal-dye detection. The decrease of absorption peak of the initial complex of yttrium(III) and 4-(2-pyridylazo)resorcinol (PAR) at 546 nm is, similarly as in the case of Fe^3+^ in aforementioned detection with Wade reagent, due to the transformation of Y^3+^ ions to more stable inositol phosphate complex, thus providing the negative response of the spectrophotometric detector and low detection limits (~1 µmol levels). This method has been applied in a number of inositol phosphate studies in living cells and tissues [89,90,91] and was later modified with the use of shorter columns for significantly reduced separation times (4-fold) and increased sensitivity (10-fold) by Schlemmer et al. [92] (2001) and Guse et al. [93] (1995), respectively. The main issue of the method remains the interference of yttrium(III)-PAR complex in the presence of multivalent metals, which need to be minimized with the use of eluents of the highest purity. Post-column reaction was also used in the work of Skoglund et al. [94] (1997) who applied the complexation with Fe^3+^ ions (in HClO_4_ solution) and UV-detection at 290 nm according to previous method of Phillippy and Bland [95] (1988). In 2003 Phillippy et al. [96] reported also the use of evaporative light-scattering detection of phytic acid after the ion-chromatographic separation on the AS7 column and separation with HNO_3_ eluent. However, the detection limit of this method was twice higher (1 µg) compared to the commonly used spectrophotometric UV-detection with Fe^3+^ complex (0.5 µg).

In 1998, Skoglund et al. [97] investigated the separation of inositol phosphates with six types of strong anion-exchange columns and found the most efficient separation by Omni Pac PAX-100 using *two* separate systems. First system was employed primarily for the analysis of InsP2–InsP6 and isomers of InsP4–InsP5 using HCl eluent, while second system enabled the determination of isomers of InsP1–InsP3 collected from the selected elution fractions of the first system. In the second part, separation was achieved using NaOH eluent and a conductometric detection was applied in combination with continuous regeneration of suppressor with H_2_SO_4_, based on the previous work of Smith and MacQuarrie [98] (1988). A similar ion-exchange chromatographic method was later used also for the determination of inositol phosphates and other biologically important anions in rat brain [99] as well as for determination of phytic acid in millet and cowpea seeds [100]. In comparison to previous HPLC methods, the use of suppressed conductivity detection notably improved the sensitivity (limit of detection ~0.3 µM) and reported RSD values, whilst the separation with alkaline hydroxide eluate reduced the retention times of higher inositol phosphates and thus shortened also the total analysis time.

Another interesting detection technique, that was introduced in the same period by Skoglund et al. [101] (1997), is the pulsed amperometric detection (PAD) which is based on the oxidation of carbohydrate –CHOH group to –C=O and resulting anodic current measured on the gold working electrode in a flow-through 3-electrode cell. Contrary to the conductivity detection, which exhibits a higher response for analytes with greater absolute charge (i.e., higher inositol phosphates), the PAD detector gives increasing sensitivity with decreased number of phosphate groups and is thus useful only for determination of inositol mono- and di-phosphate isomers (and clearly for the inositol detection as well). The limits of detection were 0.04 and 0.4 pmol for InsP1 and InsP2, respectively, which is 10–100 times lower from the other reports for lower inositol phosphates [94].

Although above mentioned IC methods showed promising advances for the analysis of not only inositol phosphates with different numbers of phosphate groups, but also different isomeric forms (enantiomers are excluded), due to the slight differences in valences and structures between some isomers, the separation was still not fully satisfactory. Another important issue that has to be emphasized, was also the unavailability of commercial standards of many isomers, thus some separated chromatographic peaks remained unidentified or might were recognized inaccurately due to the identification based solely with regard to the separation sequences reported in earlier publications under different separation conditions [102,103]. Therefore, in 2003 Chen and Li [104] developed a high-performance ion chromatographic (HPIC) method which led the separation of 35 inositol phosphates into 27 peaks using a linear gradient elution program (65 min) with HCl at CarboPac PA-100 column (Dionex) and UV-absorbance detection at 295 nm after post-column complexation with iron(III). The separation was optimized using the in-house reference standard solution, produced by the non-enzymatic thermal hydrolysis of dodecasodium phytate salt at 140 °C in 2 M HCl, which can in total give 63 isomers of inositol phosphates. Excluding all enantiomers, there are theoretically 39 different inositol phosphates that can be separated with the use of ion-exchange stationary phase: 4 × InsP1, 9 × InsP2, 12 × InsP3, 9 × InsP4, 4 × InsP5, and InsP6.

Interestingly, the separation with alkaline hydroxide, based on the work of Hull and Montgomery [103] (1995) who studied corn steep water processes, showed that the retention times are not always increased with increasing number of phosphate groups (and consequently increasing negative charge), which could be explained by steric hindrances that do not allow for the equal interaction of all deprotonated phosphate groups with the anion-exchange sites on the stationary phase. Moreover, in the case of hydroxide eluent, due to relatively high valences of inositol phosphates (up to −12) at alkaline pH conditions, the column capacity can be reached rapidly, resulting in the irreproducible retention times. Therefore, the separation with HCl eluent in combination with chloride-containing salt (e.g., KCl) has proven more useful as it suppresses the deprotonation level and thus decreases effective charge (*Z*) of inositol phosphates [104]. Experimental retention factors (*k*) are in good linear relationship according to the theoretical dependence on the eluent concentration (*c*) given by the following equation [105]:

*log k* = −(*Z*/*E*) *log c* + *log I*, (1)

where *E* is the charge of eluent and the constant *I* depends on the column and eluent characteristics.

**Figure 3:**
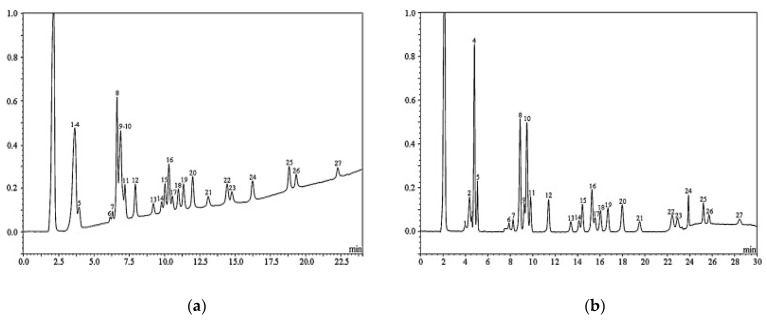
HPIC anion-exchange chromatogram of a reference sample separated using (**a**) gradient elution with HCl on CarboPac PA100 column, and (**b**) gradient elution with MSA on CarboPac PA1 column. Peak numbers correspond to the following isomers of inositol phosphates: (1–5) InsP2, (6) Ins(1,3,5)P3, (7) Ins(2,4,6)P3, (8–11) InsP3, (12) _DL_-Ins(1,5,6)P3, (13) _DL_-Ins(4,5,6)P3, (14) Ins(1,2,3,5)P4, (15) _DL_-Ins(1,2,4,6)P4, (16) _DL_-Ins(1,2,3,4)P4, (17) Ins(1,3,4,6)P4, (18) _DL_-Ins(1,2,4,5)P4, (19) _DL_-Ins(1,3,4,5)P4, (20) _DL_-Ins(1,2,5,6)P4, (21) Ins(2,4,5,6)P4, (22) _DL_-Ins(1,4,5,6)P4, (23) Ins(1,2,3,4,6)P5, (24) _DL_-Ins(1,2,3,4,5)P5, (25) _DL_-Ins(1,2,4,5,6)P5, (26) Ins(1,3,4,5,6)P5, (27) InsP6. Reprinted with permission from Elsevier [113].

The method was later applied also for determination of InsP5 and InsP6 in 6 different types of nuts and 15 dry beans by Chen [106] (2004). As an example of extensive investigation of phytic acid in food it is worth mentioning the work of Harland et al. [107] (2004) who applied the previous HPIC method [84] for the analysis of phytate and its molar ratio with Zn in 82 different types of foodstuffs using a common HCl extraction, separation with strong anion-exchange column, and detection with photo diode array (PDA) detector at 500 nm after post-column reaction and decreased absorbance of iron(III) sulfosalicylic acid complex (Wade reagent) [83]. The nutritional importance of phytate-to-zinc molar ratio and consequent medical problems due to zinc deficiency were demonstrated also by Pourghasem et al. [108] (2005). Another interesting IC application for food analysis was reported by Sekiguchi et al. [109] (2000) who developed a method which allowed not only for simultaneous inositol phosphates determination but also for the analysis of inorganic phosphates, such as orthophosphate (P), pyrophosphate (P2), trimetaphosphate, and other polyphosphates (Px). According to previously reported sample pretreatment by Shintani and Dasgupta [110] (1987) trichloroacetic acid was used for extraction and in certain cases, samples (such as cheese) were additionally purified by the cation-exchange for the removal of Ca^2+^ ions as the peak distortion and retention time shortening was observed for tetrapolyphosphate (P4) and phytic acid. Separation was performed with KOH eluent on a high-capacity IonPac AS11 column (Dionex) which showed better characteristics than the PAX-100 column previously used by Matsunaga et al. [111] (1998). With the use of on-line hydroxide eluent generator, the precision of the method (RSD = 1.8%) was significantly improved in comparison to the elution with off-line prepared hydroxide eluent (RSD ~ 6–10%) owing to significantly higher purity of KOH, which is achieved mainly because of minimized carbonate dissolution [112]. A different type of HPIC separation improvement was presented in 2010 by Blaabjerg et al. [113] who applied the gradient elution by methanesulfonic acid (MSA) on CarboPac PA1 column (Dionex) for determination of InsP2–InsP6 in pig food (wheat, soybean, rapeseed cake) and gastric and ileal digesta samples [114]. In comparison to the HCl elution, the use of MSA eluent resulted in almost horizontal baseline (see Figure 3), making the integration and thus quantification of inositol phosphates considerably more precise, as well as extending the absorbance range of UV-detection at 290 nm after post-column reaction with Fe^3+^ in HClO_4_. The method allowed for separation of 23 of total 27 investigated isomers of inositol phosphates and, due to the limited commercial availability of InsP2–InsP5 standards, the quantification was performed using the correction factors obtained with the inductively coupled plasma (ICP) analysis of total phosphorous content. The reported limit of detection for InsP2–InsP6 was 0.9–4.4 mg/L phosphorous.

In the same period mass spectrometry (MS) was applied for the detection of inositol phosphates as reported by Liu et al. [115] (2009) who developed an anion-exchange chromatography coupled to tandem mass spectrometry (HPIC-MS/MS) method for simultaneous analysis of Ins–InsP6 in a complex biological matrices, based on the previous method for inositol analysis [116]. Analytes were identified by selective reaction monitoring using a triple quadrupole mass spectrometer in negative ion electrospray ionization (ESI) mode and adenosine 5′-monophosphate was used as an internal standard for quantification with detection limit of 0.25 pmol for all inositol phosphates. MS detection was also applied by Sun and Jaisi [117] (2018) for the identification of oxygen isotope (δ^18^O) signals using the isotope ratio mass spectrometry (IRMS) method for the study of distribution of inositol phosphates in feed ingredients for selected ruminant and non-ruminant animals and their excreta. The fractionation factor is calculated on the basis of the difference between oxygen isotope value of the incorporated vs. ambient water oxygen that takes place during the enzymatic degradation of phytate as reported by von Sperber et al. [118] (2015). More recently, tandem mass spectrometry was coupled also with hydrophilic interaction liquid chromatography (HILIC-MS/MS) for the analysis of InsP6 and InsP7 in mammalian cells (human blood cells, HEK293, and mouse brains) with the LOD values of 5 and 2 pmol, respectively, as reported by Ito et al. [119] (2018), who used a separation with ammonium formate and ammonium carbonate buffer on HILICpac VG-50 column.

### 3.3. Gas Chromatography (GC)

First reports of gas chromatography applications for the analysis of phytic acid can be found around 1985. Due to high boiling points of inositol phosphates, preliminary hydrolysis for the removal of phosphate groups and subsequent derivatization of the resulting inositol is required prior to separation procedures generally performed on the capillary column. The derivatization can be accomplished using different reagents, namely: heptafluorobutyrylimidazole [120], trifluoroacetic anhydride [121] and trimethylchlorosilane [122]. The latter was used also by de Koning [123] (1994) for producing hexa-*O*-trimethylsilyl ether according to the work by Roberts et al. [124] (1965) and an addition of scyllitol (*scyllo*-inositol), the geometric isomer of *myo*-inositol, as an internal standard for quantification of phytic acid in cereals and pet foods. The GC method was further improved also for the analysis of biological samples, such as plasma and urine, by March et al. [125] (2001) who used mass spectrometry detection on the basis of the previous GC-MS method [126]. Another modification was introduced in the work by Park et al. [127] (2006) who applied the gas chromatographic separation method with the flame ionization detector (GC-FID) for the analysis of phytic acid levels in infant foods after derivatization procedure using the mixture of hexamethyldisilazane (HMDS) and chlorotrimethylsilane (TMCS). A comparison of the GC-FID results with the HPLC-RI, colorimetric AOAC assay with preliminary ion-exchange purification [128], and spectrophotometric analysis using Wade reagent is given in the publication as well. The spectrophotometrically determined values showed significantly higher levels than those of chromatography which is due to the presence of lower inositol phosphates in the samples as discussed before [129].

### 3.4. Thin Layer Chromatography (TLC)

Contrary to the numerous publications of LC and IC applications, only few reports of the use of thin layer chromatography for the analysis of inositol phosphates can be found in the literature. In 1957 Schormüller and Würdig [130] developed a paper chromatographic method, which was later applied also to thin layer cellulose plates but failed to separate InsP5 isomers. In 1969 Angyal and Russell [131] used methylation and separation of the resulting methyl esters of inositol phosphates on both silica gel and alumina, while Emilsson and Sundler [132] (1984) performed the separation using polyethyleneimine (PEI) cellulose plates and visualization of the spots with salicylsulfonic acid-ferric chloride procedure [133]. The method was later improved for the processing of a larger sample series (~80/day) by Hatzack and Rasmussen [134] (1999) using the cellulose precoated glass plates and detection with acidic molybdate reaction followed by the heating and UV-light exposure (254 nm) which resulted in visualization of faint blue spots, as originally described by Bandursky and Axelrod [135]. Although some other applications of TLC for the studies of InsPs followed in the next years, such as the work by Shi et al. [136] (2005), the use of the method is limited due to the low separation capacity which requires additional pretreatment of complex (biological) samples in order to remove interfering nucleotides, and co-isolated phosphosugars have to be identified by specific detection. Moreover, for the structural confirmation of identified inositol phosphates, the use of isomer specific methods, such as HPLC, HPIC, and/or NMR spectroscopy, remains mandatory.

### 3.5. Electrophoresis

The first application of paper ionophoresis for the separation of inositol phosphates was reported in 1956 by Arnold [137], however the method had no advantage over the paper chromatographic methods at the time as it was unable to separate InsP5 from InsP6. The improvement of the method followed by Seiffert and Agranoff [138] (1965) with the use of high-voltage paper electrophoresis (HVPE) employing oxalate buffer for the separation of inositol phosphates from hydrolysates of rat tissues, and by Tate [139] (1968) who modified the procedure by moving the paper through a cooling tank of carbon tetrachloride during the electrophoresis. This method enabled the separation of all 4 isomers of InsP5 over a considerable length of paper using a moderate voltage. Paper electrophoresis was later used also for the analysis of phytate in pollen extracts in combination with NMR spectroscopy by Jackson et al. [140] (1982).

Development of other electrophoresis methods followed with the use of capillary isotachophoresis (cITP) which was used by Kikunaga et al. [141] (1985) to determine the phytate content in rice, rye, wheat, and barely samples, while Blatny et al. [142] (1995) applied cITP for the analysis of cereal grains and legumes. The plant extracts were purified by a common precipitation with iron(III) and converted into sodium salt form by NaOH prior to electrophoretic separation of inositol phosphates and orthophosphate. In 1992 Nardi et al. [143] used a capillary zone electrophoresis (CZE) for the determination of phytate in soybeans using an indirect on-column UV-photometric detection by choosing the background electrolyte of benzoic acid/histidine mixture at pH 6.2, which allowed to complete the separation in 4 min. The reported detection limit was ~0.1 µM. The combination of both methods was applied by Prokorátová et al. [144] (2004) who coupled the online cITP with CZE and conductivity detection for the analysis of phytic acid in the meat additives as a marker of a plant source in meat products. A similar approach was used later for determination of also lower inositol phosphates in barley by Kvasnička et al. [145] (2011).

Another type of electrophoretic analysis of phytate was introduced by Losito et al. [146] (2009) who applied polyacrylamide gel electrophoresis (PAGE) for the separation of selected inositol phosphates as well as inositol pyrophosphates (InsP7–InsP13). Detection using both Toluidine reagent and 4′,6-diamidino-2-phenylindole (DAPI) demonstrated the unequivocal detection of various inositol phosphates. The method was used for the analysis of phytate content in different plants, such as tomato, rice, and tobacco by Alimohammadi et al. [147] (2013) and recently in black pepper leaves by Giridhari et al. [148] (2017). In the case of biological samples and related biochemical studies of inositol phosphates and their cellular activity, the direct analysis by PAGE is often limited due to lower concentrations of inositol phosphates and larger extraction volumes that are thus required to obtain sufficient amounts of the analytes. This difficulty was overcome by Wilson et al. [149] (2015), who developed a TiO_2_ microsphere purification/enrichment procedure based on the adsorption of inositol phosphates on the TiO_2_ beads and separation from a complex extract mixtures or diluted biofluids. A similar approach was applied for the determination of InsP6–InsP8 and nucleotides (ATP and GTP) in mammalian cell and tissue extracts, human plasma and urine, and slime molds in combination with PAGE [149], SAX-HPLC [150], capillary electrophoresis (CE) [151], and NMR techniques [152,153]. Separation with PAGE revealed the existence of additional and previously uncharacterized pyrophosphorylated inositol reaction products and therefore the likely underestimation of inositol pyrophosphates and their signalling contribution in cells when analyzed via traditional (chromatographic) techniques.

However, due to abundant inorganic polyphosphates in certain biological samples, such as yeasts, and consequently suppressed signals of inositol phosphates, some limitations with the use of PAGE apply. Improved electrophoretic approach for the analysis of inositol phosphates and inositol polyphosphates was introduced by the use of capillary electrophoresis coupled to electrospray ionization mass spectrometry (CE-ESI-MS), developed very recently by Qiu et al. [151] (2020), who applied the stable isotope labeled internal standards for the quantification of InsP1–InsP8 in yeasts, plants, and mammalian cell lines and tissues. Due to relatively low detection limits, which were reported around 50–150 nM (corresponding to 0.5–1.5 fmol of analytes), the method exhibits potential for the further investigation of inositol phosphate metabolism and its role in cell signalling. Another important advantage of electrophoresis over the other commonly used chromatographic separation methods is also the speed and generally lower running costs of the analysis.

## 4. Spectroscopy

### 4.1. UV-Vis Spectrophotometry

As mentioned before, spectrometric methods have been frequently applied for the detection of inositol phosphates in combination with both the precipitation procedures and the separation (chromatographic) techniques. However, due to the absence of the specific absorption spectra as well as colorimetric reagents, additional principles had to be applied as further reviewed in the following discussion. The use of UV-Vis spectrophotometric detection of phytic acid began early alongside the first (classical) precipitation methods, where phytate content was determined colorimetrically at 830 nm via phosphorous assay by the molybdenum blue reaction [37,39] or indirectly by determination of iron(III) in the precipitate [41] or supernatant [154] after the release from ferric phytate precipitate by NaOH reaction or acidic wet digestion. The latter can be applied also for the hydrolyzation of phytic acid and lower inositol phosphates and thus for the analysis of released phosphate, however the enzymatic approach has been used more often [155]. A similar blue-colored molybdenum complex can be formed also directly with phytate without preliminary dephosphorization as originally reported by Raheja et al. [156] (1973) and modified by Mohamed et al. [157] (1986). However, the chromogenic reagent requires the use of elementary mercury. This method has been recently applied by Santiviago et al. [158] (2020) for the determination of phytic acid in poultry wastewater with the reported detection limit of 0.18 mg/L.

Low concentrations of phytic acid can be also detected directly by the absorption peak at 290 nm which corresponds to the soluble iron(III) phytate complex, as was applied by the post-column derivation for the online detection after chromatographic separation [76]. An indirect approach is also possible by the use of the competitive complexation reaction as the metal ion is released from its initial coordination compound and complexed by stronger phytate ligand. This principle was applied using the yttrium(III) 4-(2-pyridylazo)resorcinol (PAR) complex [88] or more frequently the iron(III)-sulfosalicylic complex (Wade reagent) [79], with the detection of a decreased absorption (negative) peak at 546 or 500 nm, respectively. Wade reagent was applied also for determination of phytate in combination with the multi-pumping flow system by Carneiro et al. [159] (2002) and the analysis of different types of food samples, such as corn, soybeans, wheat, sunflower, oats, and rye, as reported also by Agostinho et al. [160] (2016).

In 1986 Harland and Oberleas [128] developed a spectrophotometric analysis of phytate using the preliminary ion exchange-purification and digestion of the samples with sulfuric and nitric acid, followed by the reaction with molybdate and 1-amino-2-hydroxynaphthalene-4-sulfonic acid reagent and detection of phosphate at 640 nm, according to the original molybdenum blue colorimetric method. This procedure was generally accepted and also published by the AOAC [161] as official analytical method for determination of phytic acid content in foodstuffs. However, due to the detection of also lower inositol phosphates, overestimated phytate content might be found by this procedure, as reported by Frølich et al. [162] (1986) and Lehrfeld and Morris [129] (1992). The method has been recently modified by McKie and McCleary [163] (2016) for a faster determination of phytate, using an enzymatic dephosphorylation by phytase that is specific for the InsP6–InsP2, and an alkaline phosphatase which ensures the release of the final phosphate from InsP1, and thus omitting the tedious acidic digestion of the sample extracts. The modified procedure was applied also for the analysis of phytic acid in rice samples with low phytic acid bioavailability by Perera et al. [164] (2019).

Another principle was introduced by Kamaya et al. [165] (1995), who developed an indirect spectrophotometric method based on the replacement complexation reaction of phytic acid, in which the (organic) ligand with absorption band is released by transformation of the metal ion into more stable phytate complex, enabling the detection of the resulting absorbance peak of free (organic) ligand. Amongst the investigated combinations of different metals (Zn, Co, Ba, Ca, Cu, Bi) and ligands (fluoranilate, chloranilate, iodoranilate), the highest sensitivity of the method was found for the zinc chloranilate complex, with the detection limit of ~3 µM. However, the presence of orthophosphate or other inositol phosphates can strongly interfere with the analysis of phytate in food products.

### 4.2. Fluorescence Spectroscopy

In comparison to UV-Vis spectroscopy, fluorimetric methods were applied rarely for the detection of inositol phosphates, however some papers can be found in the literature, starting with the work of Irth et al. [72] (1990), who used a similar competitive complexation reaction as described above. In this case Fe^3+^ ions are initially present in the weakly-fluorescent complex with methylcalcein blue (MCB) and transformed into more stable inositol triphosphate-complex, while the released MCB ligand exhibits strong fluorescence signal in its “free” form allowing for the determination of lo-ppb concentration levels of InsP3. The first procedure of fluorimetric determination of phytic acid was developed by March et al. [166] (1999) by applying the activation effect of phytate on the oxidation of 2,2′-dipyridyl ketone hydrazine catalyzed by copper(II) ions, which results in the highly fluorescent reaction product and low detection limit (0.03 mg/L). The method was applied for the determination of phytate in human urine and 9 different types of food samples. Another example of competitive complexation was reported by Chen et al. [167] (2007) who applied the fluorimetric replacement reaction, in which phytate ligand replaced Cu^2+^ ion from its initial gelatin complex, liberating the fluorescent gelation molecule. However, higher detection limit (0.23 mg/L) was obtained by this method for the analysis of urine samples. A similar principle was later applied by Cao et al. [168] (2011) where phytic acid efficiently “catches” Cu^2+^ ion from the previously prepared complex with 2,2′-bipyridine complex, releasing the fluorescent ligand and allowing for improved limit of detection (0.12 mg/L). In 2014, Kolozsvari et al. [169] discovered that similarly as inorganic polyphosphates both InsP6 and InsP5 induce the shift of DAPI fluorescence peak which is emitted at approximately 550 nm after excitation with light of wavelength 415 nm. On the other hand, lower inositol phosphates, such as InsP4 and InsP3, are unable to cause the shift. This method was applied for the investigation of enzymatic activity of inositol polyphosphate multikinase and the determination of InsP6 in plant seeds, based on the absence of inorganic polyphosphate in the investigated sample matrix, as confirmed by the use of Raman spectroscopy [170].

A synchronous fluorescence analysis of phytate was introduced by Chen et al. [171] (2009), who applied the formation of a ternary complex between Fe^3+^ ion, phytic acid, and 1,10-phenanthroline. Using the synchronous luminescence spectroscopy, the obtained signal intensity was enhanced proportionally to the increased phytate amount in a wide concentration range (0.33–32 mg/L), however the improvement of the selectivity is needed for the analysis of food samples. Recently, the fluorimetric detection was applied also for the development of a highly sensitive phytic acid sensor, using the glutathione-functionalized graphene quantum dots (GQD@GSH), as reported by Qu et al. [172] (2018). The “off-on” fluorescence method is based on the strong chelation ability of phytate which removes the quenching effect of Fe^3+^ ions and results in restored fluorescence of the quantum dots allowing for the determination of low phytic acid levels (14 nM) in corn and human serum samples.

### 4.3. Nuclear Magnetic Resonance (NMR)

Because of the presence of phosphorous in phytic acid and lower inositol phosphates, ^31^P NMR spectroscopy has been frequently applied independently or in combination with (separation) methods for the analysis of biological samples, as well as for the studies of hydrolysis paths of phytic acid [162] and its interactions with metal ions [173,174]. The main advantage of NMR technique is the ability to distinguish not only structural isomers of InsP1–InsP6 but also their stereoisomers and it can be applied even without complex sample preparation. The first application of NMR was reported in 1980 by O’Neil et al. [175] who applied ^31^P Fourier transformation for the determination of phytate in a variety of foodstuffs after the separation from free phosphate by modification of ion-exchange method [176]. A strong dependence of the chemical shifts on the pH value was also well demonstrated in the paper. NMR was applied in combination with paper electrophoresis for the determination of phytate in pollen by Jackson et al. [140] (1982). In comparison to the ferric precipitation method, which was commonly used at the time, Frølich et al. [162] (1986) used the NMR for the determination of phytate in whole grains and for the investigation of stepwise enzymatic hydrolysis pathway of phytic acid.

A modification of the method followed by the work of Mazzola et al. [177] (1986) who eliminated the food matrix effects by the use of standard addition instead of an internal standard, and removed the NMR signal interferences due to paramagnetic ions by the addition of strong metal-complexing agent EDTA. Another major improvement was presented by Johnson et al. [178] (1995) who introduced the two-dimensional total correlation spectroscopy (2D-TOCSY) NMR technique which allows simultaneous determination of the structures of multiple inositol phosphates in a mixture without separation step. Many applications were later developed also for the analysis of inositol phosphates in soils by a single-step alkaline extraction and NMR detection procedure as reviewed by Turner et al. [179] (2007). However, obtaining accurate quantitative results might be a difficult task due to the problematic peak identification, which is complicated by variations in chemical shift with pH and ionic strength, and quantification of NMR signal for individual species in the crowded and overlapping phosphate monoester region. Although spectral deconvolution techniques have been applied for this purpose, the overestimation of phytate and other inositol phosphates can be found by NMR as discussed by Doolette et al. [180] (2011). New insights regarding the mechanism and pathway of phytate dephosphorylation were obtained by Wu et al. [181] (2015), who applied NMR analysis in combination with the oxygen isotope ratio studies and HPLC separation, and more recently by Watson et al. [182] (2019) who studied the thermal degradation of phytate using different 2D ^31^P and ^1^H NMR techniques, which provide vital information on the relative position of nuclei. Furthermore, NMR was applied also in combination with metabolic ^13^C-labeling of inositol compounds ([^13^C_6_]-Ins, [^13^C_6_]InsP5, [^13^C_6_]InsP6, and [^13^C_6_]InsP7), which enables the detection and quantification of the analytes within complex cell extracts and at physiological concentration levels as reported by Harmel et al. [153] (2019). This procedure is based on the improved enzymatic conversion of [^13^C_6_]glucose to [^13^C_6_]inositol, which is introduced to mammalian cell lines, and a subsequent detection of the in vivo generated [^13^C_6_]-InsP species without a separation or enrichment procedure. The method has been used also for the biochemical characterization of enzymatic activity of inositol hexakisphosphate kinase 1 (InsP6K1) in real time.

Although very useful information can be derived from the NMR analysis of (stereo)isomers of InsP1–InsP6, due to relatively expensive laboratory equipment and in-depth knowledge that is required for the interpretation of the rather complicated signals, NMR has not been the method of choice for the routine analysis of phytic acid and other inositol phosphates.

### 4.4. Inductively Coupled Plasma (ICP)

Particularly in the case of routine analysis of biological fluids in clinical laboratory, the determination of inositol phosphates by ICP shows some advantages, owing to usually simpler and faster analytical procedures and lower detection limits in comparison to more complexed and time-consuming chromatographic methods. The first publication of the use of ICP was reported in 1991 by Plaami and Kumplulainen [183] where phytic acid was first precipitated by ferric reaction and determined quantitatively as phosphorous by ICP-atomic emission spectrometry (ICP-AES) after conversion of complexed iron to ferric hydroxide and consequent release of phytate by NaOH. The method was applied for the analysis of phytic acid content in oat samples [184]. A different approach was introduced by Grases and Llobera [185] (1996) who used the ICP-AES determination of phosphorous after previous separation of synthetic and human urine samples by anionic resin (AG1-X8). Taking into account the common phytic acid amount in urine (~0.4 g/L) and detection limit for phosphorous by the ICP-AES (0.5 g/L), a twenty-fold pre-concentration of phytate from eluate was recommended by the authors. In 2004 Grases et al. [186] developed a fast ICP-AES method for a routine determination of phytic acid in urine based on direct phosphorous analysis. Due to the presence of orthophosphate in the matrix, purification of the sample is crucial and was achieved using anion-exchange resin and two eluents, namely HCl for the removal of orthophosphate in the first step and HNO_3_ for the subsequent elution of phytate. However, other inositol phosphates can also be present in the sample matrix but their concentration levels in urine are neglectable. The reported limit of detection was 64 µg/L of phytic acid.

Sensitivity of ICP analysis of phytate was improved with use of mass spectrometry (MS) detection of ^31^P by Muñoz and Valiente [187] (2003) who demonstrated its applicability even for low amounts of biological samples with reported limit of detection 5 µg/L of phytic acid. The method was further optimized by Muñoz et al. [188] (2010) for the analysis of urine with the use of off-line anion exchange solid phase extraction (SPE) cartridge packed with aforementioned anion-exchange AG 1X8 resin. The authors report no need of pre-concentration step even for low amounts of samples (1 mL) due to the low detection limits of ICP-MS. In the case of food analysis Liu et al. [189] (2017) introduced a microwave accelerated extraction procedure which, in comparison to traditional solid-solvent extraction method, showed better characteristics by significantly reducing the optimal extraction time from typical 3 h to 10 min.

## 5. Sensors

### 5.1. Electrochemical Biosensors

The majority of sensor applications for the detection of phytic acid were developed in the last two decades and were mostly based on the use of materials capable of a molecular recognition, such as enzymes used in various types of biosensors. One of the first was an electrochemical biosensor with a sequentially acting phytase and pyruvate oxidase enzymes which were co-immobilized on the Pt working electrode with the use of polycarbamoyulsulfonate hydrogel, as reported by Mak et al. [190] (2004). Phytic acid is first hydrolyzed and the resulting orthophosphate enables the enzymatic production of H_2_O_2_ and amperometric detection at 0.6 V vs. Ag/AgCl reference electrode with the limit of detection 2 µM. In the work of Caseli et al. [191] (2006) cyclic voltammetry was used for the detection of phytate on the ITO electrode with a 35-layer of a mixed phytase-lipid biocatalytic film produced by the Langmuir–Blodgett technique [192]. However, the detection limit was 10-times higher than in the previously mentioned method. In the following years many different techniques were examined for the immobilization of enzymes on the solid surface, including the deposition of layer-by-layer (LbL) film of phytase alternated with poly(allylamine) hydrochloride on the ITO substrate covered with Prussian Blue, used for amperometric detection of phytic acid [193]. A similar approach was later applied by Moraes et al. [194] (2010) also in combination with impedance spectroscopy which improved the sensitivity of the method (LOD was ~0.4 µM). However, the study showed the importance of nonspecific interactions, such as electrostatic cross-linking, which might even surpass the specific interactions of highly charged phytate analyte under given conditions and thus reduce the selectivity and applicability of the impedance spectroscopy for determination of phytate. With the use of square wave voltammetry in combination to fast Fourier transform (FFT-SWV) Esmaeili et at. [195] (2019) developed a CeO_2_ nanoparticles modified glassy carbon sensor for detection of low concentrations of phytic acid, which is based on the decreased oxidation (anodic) peak of [Fe(CN)_6_]^3−/4−^ redox probe upon the increasing concentration of phytate.

### 5.2. Fluorescence Nanoprobes

As reported by of Shi et al. [196] (2019), low detection limit (10 µM) is obtained also by the fluorescent nanosensor (or nanoprobe) based on conjugated polyelectrolyte dots fabricated from a polymer with π-delocalized backbone bearing meta-substituted pyridyl groups which allow complexation of Fe^3+^ ions and thus lead to fluorescence quenching. With the addition of phytic acid, Fe^3+^ is transformed into more stable phytate complex, resulting in the fluorescence signal at 444 nm. More recently Rodrigues et al. (2020) presented a proof of concept for the spectrophotometric detection of phytic acid based on the thiophene/fluorene copolymer Langmuir monolayer as the matrix for immobilization of phytase without provoking significant loss of enzymatic activity. Upon the hydrolysis of phytate, the produced orthophosphate is monitored at 830 nm as a blue molybdate complex as described before [161]. However, the detection based on the enzymatic products of phytate dephosphorylation can be accompanied by poor selectivity, particularly when lower inositol phosphates or other phosphate containing biomolecules are present in the samples as well. Therefore, Lee et al. [197] (2014) synthesized a tetranaphthoimidazolium receptor, which shows a selective fluorescence enhancement for InsP6 over InsP3, orthophosphate, pyrophosphate, AMP, ADP, and ATP at 465 nm with reported limit of detection 0.23 µM. This fluorescence chemosensory was also applied for the first imaging of phytate in a live cell.

It is worth mentioning that due to excellent adsorption properties of phytic acid and its strong binding interactions with several types of electrodes and nanomaterials, it has also been used for the fabrication of different sensors for detection of other analytes. In 2010 Wang et al. [198] applied phytate together with Au nanoparticles for the construction of H_2_O_2_ sensor an electron-conductive 3D mesoporous film by means of LbL technique. In the last decade, many applications of other phytic acid-fictionized nanomaterials (graphene oxide, TiO_2_, SiO_2_, glassy carbon) have been used for the detection of various heavy metals [199] and biomolecules, such as dopamine [200] and glucose [201].

## 6. Conclusions

From the first simple gravimetric methods, developed more than a century ago, to the novel bio- and nano-sensor application, the determination of phytic acid was never an easy analytical task. This is mostly due to (i) intriguing chemical properties of phytates and interferences of accompanying lower (and to certain extend also higher) inositol phosphates and other hydrolyzation products, such as inorganic phosphate, (ii) absence of characteristic signals (e.g., absorbance), as well as (iii) strong interactions with most of metal cations and biomolecules that are generally present in the sample matrix. The development of analytical methodologies that have been applied over the years are discussed in the present work for a systematical and chronological overview. Table 1 summarizes the most significant analytical contributions and corresponding references for the determination of phytic acid and other inositol phosphates.

Depending on the research field, the most commonly used sample preparation is still the acidic extraction of inositol phosphates from preliminary dried and ground samples. This is generally performed with HCl by the traditional 2–3 h solid-solvent extraction method, however the extraction times can be significantly reduced (down to 10 min) by the use of microwave accelerated extraction procedure. As stressed out, determination of phytic acid and/or inositol phosphates usually requires a sufficient separation of analytes, which can be performed by different types of chromatographic and electrophoretic techniques. The most success has been obtained by the use of high-performance ion-exchange chromatography (HPIC), which allows the separation of most of isomers of inositol phosphates, excluding the stereoisomers. Different types of detection techniques can be applied, including the most commonly used suppressed conductivity detection which exhibits better characteristics over the spectrophotometric detection after the post-column reactions, usually based on the iron(III) complexation mechanism (Wade reagent) or molybdate blue reaction. The latter was accepted also as an official AOAC method and is the most commonly applied reagent for the spectroscopic determination of phytate, based on the classical molybdenum blue reaction from 1922 via phosphorous assay after enzymatic hydrolysis of phytate. For routine analysis of phytic acid in samples in medicine, such as human plasma and urine, the total phosphorous determination with ICP techniques proved useful due to fast speed of analysis and simple sample pretreatment. Hereby is it important to note, that with the application of detection method that is used without preliminary purification and/or separation step(s), a special attention should be given to the potential presence of other inositol phosphates or inorganic phosphates, which can consequently lead to overestimation of the obtained values. Particularly in the case of biological investigations, such as cell signalling, the detection of different (stereo)isomers of inositol phosphates is permitted by the use of modern two-dimensional NMR techniques, however, a relatively sophisticated instrumentation is required, thus limiting the accessibility and use of this kind of detection for routine experiments. With the use of highly sensitive bio- and nano-sensors, based mainly on the fluorescence and voltammetric detection in combination with enzymatic dephosphorylation of phytate, new methods with lower LODs and ability to detect phytic acid also in the living cells have been developed in the last years. Considering the increasing number of recently published sensor applications, this will presumably be also the main field of the development of new analytical methods for determination of phytic acid and related inositol phosphates in the near future.

## Figures and Tables

**Table 1:** Summary of the main analytical methods for determination of phytic acid and other inositol phosphates arranged by methodology, separation and/or detection system, analyte(s), sample material and preparation, limit of detection (LOD) ^1^, year, and corresponding references.

Methodology	Detection	Analyte	Sample Material	Sample Preparation	LOD ^1^ (S/N = 3)	Year, Ref.
Precipitation titration (Fe^3+^)	Endpoint color indication (NH_4_SCN)	InsP6	Cereals	HCl extraction	0.3 mg/g	1914 [33]
Precipitation (Fe^3+^), digestion (Kjeldahl)	Colorimetric as PO_4_^3−^ (molybdenum blue)	InsP6	Dried food	HCl extraction	0.1 mg/g ^2^	1935 [37]
Precipitation (Fe^3+^)	Colorimetric by residual Fe^3+^ (2,2′-bipyridine)	InsP6	Soya, cereals	HCl extraction	2 µg/g	1983 [43]
Differential titrimetric standardization (OH^−^)	Potentiometric by derivatives (∂pH/∂*n*_OH-_)	InsP6	Phytate salt	Cation-exchange chromatography	1 µmol	2015 [50]
Ion-pair HPLC (C18 RP column)	Refractive index	InsP3–InsP6	Legumes	Anion-exchange chromatography	100 mg/L	1995 [66]
Ion-pair HPLC (10-SAX column)	Fluorescence by isotopic labeling (^32^P)	InsP1–InsP4, phosphates	Cultured cells	Charcoal pretreatment	0.2 nmol	1989 [71]
HPLC (10 SAX WCS)	Radioactivity by enzymatic ^32^P-labeling	InsP6	HeLa cells, rat tissue, serum, etc.	Trichloroacetic acid extraction, centrifugation	0.25 pmol (5 nM)	2008 [73]
HILIC (HILICpak VG-50 column)	Tandem mass spectrometry (HILIC-MS/MS)	InsP6, InsP7	Human blood, HEK293, mouse brain	Anion-exchange chromatography	2 pmol	2018 [119]
IC anion-exchange (SAX column)	Colorimetric (post-column reaction by Wade reagent)	InsP2–InsP6	Plants	HCl extraction	0.2 mg/g	1993 [84]
IC anion-exchange (Mono Q HR 5/5)	Colorimetric as PO_4_^3−^ (post-column enzymatic hydrolysis)	InsP2–InsP4 stereoisomers	Rat tissue	HClO_4_ extraction, charcoal	1 nmol	1986 [87]
IC anion-exchange (Mono 1, HCl elution)	Colorimetric (post-column reaction by Y^3+^-PAR)	InsP1–InsP6 stereoisomers	Cultured cells, tissue	HClO_4_ extraction, charcoal	0.1 µmol	1995 [93]
IC anion-exchange (PAX-100, HCl & NaOH elution)	UV (post-column reaction by Fe(NO_3_)_3_); Suppressed conductometry	InsP1–InsP6, isomers of InsP4–InsP5	Food, digesta	HCl extraction, ion-exchange purification	0.5 µg	1998 [97]
IC anion-exchange (AS7)	Evaporative light scattering	InsP6	Roots, tubers	HCl extraction	1.0 µg (30 µg/g)	2003 [96]
IC anion-exchange (CarboPac PA-10, NaOH elution)	Pulsed amperometric detection (PAD)	Ins, InsP1–InsP2 (isomers)	Food, digesta	HCl extraction, ion-exchange purification	0.04 pmol	1997 [101]
IC anion-exchange (CarboPac PA-100, HCl/KCl elution)	UV (post-column reaction by Fe(NO_3_)_3_)	InsP2–InsP6 (27 isomers)	Nuts, beans	HCl extraction, (solid phase cartridge)	2 µM	2003 [104,106]
IC anion-exchange (AS-11, online-generated KOH elution)	Suppressed conductometry	InsP3, InsP6, (poly)phosph-ates (P1–P52)	Ham, fish, cheese	Trichloroacetic acid extraction, cation-exchange	0.15 µM	2000 [109]
IC anion-exchange (PA-1, online MSA elution)	UV (post-column reaction by Fe(NO_3_)_3_), ICP-MS (corr. factors)	InsP2–InsP6 (23 isomers)	Wheat, soybean, digesta	HCl extraction, cation-exchange purification	0.1 mg/L	2010 [113]
IC anion-exchange (Biobasic AX, (NH_4_)_2_CO_3_ elution)	Tandem mass spectrometry (ESI-MS/MS)	InsP1–InsP6, Ins	Nut, grain, cultured cells	Acetic acid & hexane extraction	0.1 pmol	2009 [115]
IC anion-exchange (CarboPac PA-100, HCl elution)	Isotope ratio-mass spectrometry (δ^18^O IRMS)	InsP2–InsP6, phosphate	soy, corn, animal excreta	HCl extraction, anion-exchange purification	20 µg/g	2018 [117]
GC (hydrolysis, derivatization by trimethylsilyl)	Mass spectrometry (MS)	InsP6	Rat tissue, urine, plasma	Anion-exchange purification	9 µg/L	2001 [125]
GC (hydrolysis derivatization by HMDS, TMCS)	Flame ionization (FID)	InsP6	Infant food (flour and paste)	Anion-exchange purification, hexane extraction	4 ng	2006 [127]
TLC - Thin layer chromatography (cellulose glass plates)	UV (reaction by molybdate reagent)	InsP1–InsP6, organic phosphates	Barley grains	EDTA, diethyl ether, charcoal	0.1 nmol	1999 [134]
cITP/CZE - Capillary isotachophoresis/Zone electrophoresis	Conductivity detection	InsP6, phosphate	Barley, meat additives	HCl extraction	20 µg/L	2004 [144,145]
PAGE - Polyacrylamide gel electrophoresis	Toluidine blue, DAPI	InsP4–InsP13,	Tomato, rice, tobacco	HCl extraction, centrifugation	0.5 nmol	2009 [146,147]
PAGE - Polyacrylamide gel electrophoresis	Toluidine blue	InsP6–InsP8, nucleotides	Mammalian cells, tissue, plasma	TiO_2_ beads purification	0.25 nmol	2015 [149]
Spectrophotometric (AOAC method)	Colorimetric as PO_4_^3−^ (acidic/enzymatic hydrolysis)	InsP6	Various foodstuffs	Ion-exchange purification	0.9 mg/g	1986 [128]
Fluorimetric	Cu^2+^ catalyzed oxidation of 2,2-dipyridyl ketone	InsP6	Oat, wheat, grape, almond	HCl extraction, anion-exchange purification	30 µg/L	1999 [166]
NMR	2-dimensional techniques (^31^P, ^1^H)	InsP1–InsP6 stereoisomers	Soils	Alkaline extraction	3 µg/g	2007 [179]
ICP-AES	Atomic emission spectroscopy	InsP6	Urine	Anion-exchange purification	64 µg/L	2004 [186]
ICP-MS	Mass spectrometry	InsP6	Walnuts	Microwave extraction (H_2_SO_4_/HCl)	5 µg/L	2017 [189]
Enzymatic sensor	Amperometry	InsP6	InsP6 standard	Not required	1 mg/L	2004 [190]
Enzymatic sensor (CeO_2_NPs, graphene oxide, glassy carbon)	FFT-SWV (Fast Fourier transform-Square wave voltammetry)	InsP6	Corn flour	Not reported	0.07 ng/g	2019 [195]
Fluorescence nanoprobe	Fluorescence quenching (Fe^3+^, polyelectrolyte dots)	InsP6	Live cells	Digestion (trypsin)	7 µg/L	2019 [196]

^1^ If not directly reported in the reference, LOD was calculated, (e.g., from calibration curve). When possible, values were converted to comparable units, depending on the sample type (solid or liquid) and analytes. ^2^ Calculated according as the lowest reported content in the corresponding reference.

## References

[1] Torres J., Veiga N., Gancheff J.S., Domínguez S., Mederos A., Sundberg M., Sánchez A., Castiglioni J., Díaz A., Kremer C. (2008). Interaction of myo-inositol hexakisphosphate with alkali and alkaline earth metal ions: Spectroscopic, potentiometric and theoretical studies. J. Mol. Struct..

[2] Brigando C., Mossoyan J.C., Favier F., Benlian D. (1995). Conformational preferences and protonation sequence of myo-inositol hexaphosphate in aqueous solution; potentiometric and multinuclear magnetic resonance studies. J. Chem. Soc. Dalt. Trans..

[3] Veiga N., Torres J., MacHo I., Gómez K., González G., Kremer C. (2014). Coordination, microprotonation equilibria and conformational changes of myo-inositol hexakisphosphate with pertinence to its biological function. Dalt. Trans..

[4] Irvine R.F., Schell M.J. (2001). Back in the water: The return of the inositol phosphates. Nat. Rev. Mol. Cell Biol..

[5] Maga J.A. (1982). Phytate—Its chemistry, occurrence, food interactions, nutritional significance, and methods of analysis. J. Agric. Food Chem..

[6] Reddy N.R., Sathe S.K., Salunkhe D.K. (1982). Phytates in legumes and cereals. Adv. Food Res..

[7] Graf E. (1983). Applications of phytic acid. J. Am. Oil Chem. Soc..

[8] Chatree S., Thongmaen N., Tantivejkul K., Sitticharoon C., Vucenik I. (2020). Role of inositols and inositol phosphates in energy metabolism. Molecules.

[9] Graf E. (1990). Antioxidant functions of phytic acid. Free Radic. Biol. Med..

[10] Sasakawa N., Sharif M., Hanley M.R. (1995). Metabolism and biological activities of inositol pentakisphosphate and inositol hexakisphosphate. Biochem. Pharmacol..

[11] Maffucci T., Falasca M. (2020). Signalling Properties of Inositol Polyphosphates. Molecules.

[12] Torres J., Domínguez S., Cerdá M.F., Obal G., Mederos A., Irvine R.F., Díaz A., Kremer C. (2005). Solution behaviour of myo-inositol hexakisphosphate in the presence of multivalent cations. Prediction of a neutral pentamagnesium species under cytosolic/nuclear conditions. J. Inorg. Biochem..

[13] Kumar V., Sinha A.K., Makkar H.P.S., Becker K. (2010). Dietary roles of phytate and phytase in human nutrition: A review. Food Chem..

[14] Marolt G., Gričar E., Pihlar B., Kolar M. (2020). Complex Formation of Phytic Acid With Selected Monovalent and Divalent Metals. Front. Chem..

[15] Lopez H.W., Leenhardt F., Coudray C., Remesy C. (2002). Minerals and phytic acid interactions: Is it a real problem for human nutrition?. Int. J. Food Sci. Technol..

[16] Deshpande S.S., Damodaran S. (1989). Effect of phytate onsolubility, activity andconformation oftrypsin andchymotrypsin. J. Food Sci..

[17] Lee S.-H., Park H.-J., Chun H.-K., Cho S.-Y., Cho S.-M., Lillehoj H.S. (2006). Dietary phytic acid lowers the blood glucose level in diabetic KK mice. Nutr. Res..

[18] Matyka S., Korol W., Bogusz G. (1990). The retention of phytin phosphorus from diets with fat supplements in broiler chicks. Anim. Feed Sci. Technol..

[19] Noureddini H., Malik M., Byun J., Ankeny A.J. (2009). Distribution of phosphorus compounds in corn processing. Bioresour. Technol..

[20] Rao D.E.C.S., Rao K.V., Reddy T.P., Reddy V.D. (2009). Molecular characterization, physicochemical properties, known and potential applications of phytases: An overview. Crit. Rev. Biotechnol..

[21] Sanz-Penella J.M., Tamayo-Ramos J.A., Sanz Y., Haros M. (2009). Phytate Reduction in Bran-Enriched Bread by Phytase-Producing Bifidobacteria. J. Agric. Food Chem..

[22] Nagashima T., Tange T., Anazawa H. (1999). Dephosphorylation of Phytate by Using theAspergillus niger Phytase with a High Affinity for Phytate. Appl. Environ. Microbiol..

[23] Reddy N.R., Sathe S.K. (2001). Food Phytates.

[24] Menniti F.S., Miller R.N., Putney J.W., Shears S.B. (1993). Turnover of inositol polyphosphate pyrophosphates in pancreatoma cells. J. Biol. Chem..

[25] Oomah B.D., Blanchard C., Balasubramanian P. (2008). Phytic acid, phytase, minerals, and antioxidant activity in Canadian dry bean (Phaseolus vulgaris L.) cultivars. J. Agric. Food Chem..

[26] Vucenik I., Shamsuddin A.K.M. (2003). Cancer Inhibition by Inositol Hexaphosphate (IP6) and Inositol: From Laboratory to Clinic. J. Nutr..

[27] Vucenik I., Shamsuddin A.M. (2006). Protection Against Cancer by Dietary IP 6 and Inositol. Nutr. Cancer.

[28] Marolt G., Šala M., Pihlar B. (2015). Voltammetric Investigation of Iron(III) Interactions with Phytate. Electrochim. Acta.

[29] Graf E., Mahoney J.R., Bryant R.G., Eaton J.W. (1984). Iron-catalyzed hydroxyl radical formation. Stringent requirement for free iron coordination site. J. Biol. Chem..

[30] Grases F., Llobera A. (1998). Experimental model to study sedimentary kidney stones. Micron.

[31] Jariwalla R.J., Sabin R., Lawson S., Herman Z.S. (1990). Lowering of serum cholesterol and triglycerides and modulation of divalent cations by dietary phytate. J. Appl. Nutr..

[32] Graf E. (1986). Phytic Acid: Chemistry & Applications.

[33] Heubner W., Stadler H. (1914). Über eine Titration-methode zur Bestimmung des Phytins. Biochem. Z..

[34] Rather J.B. (1917). THE DETERMINATION OF PHYTIN PHOSPHORUS IN PLANT PRODUCTS. 1. J. Am. Chem. Soc..

[35] Averill H.P., King C.G. (1926). THE PHYTIN CONTENT OF FOODSTUFFS. J. Am. Chem. Soc..

[36] Harris R.S., Mosher L.M. (1934). Estimation of Phytin Phosphorus. Ind. Eng. Chem. Anal. Ed..

[37] McCance R.A., Widdowson E.M. (1935). Phytin in human nutrition. Biochem. J..

[38] Padmanabhan C., Sundaram S. (1955). A new method for the estimation of Nitrogen in Cellulose Nitrate. Curr. Sci..

[39] Briggs A.P. (1922). A modification of the bell-doisy phosphate method. J. Biol. Chem..

[40] Nagul E.A., McKelvie I.D., Worsfold P., Kolev S.D. (2015). The molybdenum blue reaction for the determination of orthophosphate revisited: Opening the black box. Anal. Chim. Acta.

[41] Young L. (1936). The determination of phytic acid1. Biochem. J..

[42] Davies N.T., Reid H. (1979). An evaluation of the phytate, zinc, copper, iron and manganese contents of, and Zn availability from, soya-based textured-vegetable-protein meat-substitutes or meat-extenders. Br. J. Nutr..

[43] Haug W., Lantzsch H.-J. (1983). Sensitive method for the rapid determination of phytate in cereals and cereal products. J. Sci. Food Agric..

[44] Holt R. (1955). Studies on dried peas I—The determination of phytate phosphorus. J. Sci. Food Agric..

[45] Reeves R.J., Carroll R.T., Gennaro G.P. (1979). Titration of phytic acid. Talanta.

[46] Thompson D.B., Erdman J.W. (1982). Phytic Acid Determination in Soybeans. J. Food Sci..

[47] Xu P., Price J., Aggett P.J. (1992). Recent advances in methodology for analysis of phytate and inositol phosphates in foods. Prog. Food Nutr. Sci..

[48] Crea F., De Stefano C., Milea D., Sammartano S. (2008). Formation and stability of phytate complexes in solution. Coord. Chem. Rev..

[49] Burgos-Luján I., Tong A.Z. (2015). Determination of Phytic Acid in Juices and Milks by Developing a Quick Complexometric-Titration Method. Food Anal. Methods.

[50] Marolt G., Pihlar B. (2015). Potentiometric determination of phytic acid and investigations of phytate interactions with some metal ions. Acta Chim. Slov..

[51] Santiviago Petzoldt C., Peralta Lezcano J., López Moreda I. (2020). Removal of orthophosphate and dissolved organic phosphorus from synthetic wastewater in a combined struvite precipitation-adsorption system. J. Environ. Chem. Eng..

[52] Lolas G.M., Markakis P. (1975). Phytic acid and other phosphorus compounds of beans (Phaseolus vulgaris L.). J. Agric. Food Chem..

[53] Duhan A., Chauhan B.M., Punia D., Kapoor A.C. (1989). Phytic acid content of chickpea (Cicer arietinum) and black gram (Vigna mungo): Varietal differences and effect of domestic processing and cooking methods. J. Sci. Food Agric..

[54] Desjobert A., Petek F. (1956). Chromatographie sur papier des esters phosphoriques de l’inositol; application a l’étude de la dégradation hydrolytique de l’inositolhexaphosphate. Bull. Soc. Chim. Biol..

[55] Posternak S., Posternak T. (1929). Sur la configuration de l’inosite inactive. Helv. Chim. Acta.

[56] Smith D.H., Clark F.E. (1952). Chromatographic Separations of Inositol Phosphorus Compounds. Soil Sci. Soc. Am. J..

[57] Cosgrove D.J. (1963). The Isolation of Myoinositol Pentaphosphates from Hydrolysates of Phytic Acid. Biochem. J..

[58] Isaacks R.E., Harkness D.R., Froeman G.A., Sussman S.A. (1975). Studies on avian erythrocyte metabolism. I. Procedure for separation and quantitation of the major phosphorylated metabolic intermediates by anion exchange chromatography. Comp. Biochem. Physiol..

[59] Bartlett G.R. (1959). Phosphorus assay in column chromatography. J. Biol. Chem..

[60] Tangendjaja B., Buckle K.A., Wootton M. (1980). Analysis of phytic acid by high-performance liquid chromatography. J. Chromatogr. A.

[61] Camire A.L., Clydesdale F.M. (1982). Analysis of Phytic Acid in Foods by HPLC. J. Food Sci..

[62] Knuckles B.E., Kuzmicky D.D., Betschart A.A. (1982). HPLC Analysis of Phytic Acid in Selected Foods and Biological Samples. J. Food Sci..

[63] Graf E., Dintzis F.R. (1982). Determination of phytic acid in foods by high-performance liquid chromatography. J. Agric. Food Chem..

[64] Foda N.H. (1995). High performance liquid chromatographic determination of nalidixic acid in tablets. J. Liq. Chromatogr..

[65] Sandberg A.-S., Ahderinne R. (1986). HPLC Method for Determination of inositol Tri-, Tetra-, Penta-, and Hexaphosphates in Foods and Intestinal Contents. J. Food Sci..

[66] Burbano C., Muzquiz M., Osagie A., Ayet G., Cuadrado C. (1995). Determination of phytate and lower inositol phosphates in Spanish legumes by HPLC methodology. Food Chem..

[67] Lehrfeld J. (1989). High-performance liquid chromatography analysis of phytic acid on a pH-stable, macroporous polymer column. Cereal Chem..

[68] Berridge M.J., Irvine R.F. (1984). Inositol trisphosphate, a novel second messenger in cellular signal transduction. Nature.

[69] Heslop J.P., Irvine R.F., Tashjian A.H., Berridge M.J. (1985). Inositol tetrakis- and pentakisphosphates in GH4 cells. J. Exp. Biol..

[70] Morgan R.O., Chang J.P., Catt K.J. (1987). Novel aspects of gonadotropin-releasing hormone action on inositol polyphosphate metabolism in cultured pituitary gonadotrophs. J. Biol. Chem..

[71] Sulpice J.-C., Gascard P., Journet E., Rendu F., Renard D., Poggioli J., Giraud F. (1989). The separation of [32P] inositol phosphates by ion-pair chromatography: Optimization of the method and biological applications. Anal. Biochem..

[72] Irth H., Lamoree M., de Jong G.J., Brinkman U.A.T., Frei R.W., Kornfeldt R.A., Persson L. (1990). Determination of d-myo-1,2,6-inositol trisphosphate by ion-pair reversed-phase liquid chromatography with post-column ligand exchange and fluorescence detection. J. Chromatogr. A.

[73] Letcher A.J., Schell M.J., Irvine R.F. (2008). Do mammals make all their own inositol hexakisphosphate?. Biochem. J..

[74] Azevedo C., Saiardi A. (2006). Extraction and analysis of soluble inositol polyphosphates from yeast. Nat. Protoc..

[75] Laha D., Johnen P., Azevedo C., Dynowski M., Weiß M., Capolicchio S., Mao H., Iven T., Steenbergen M., Freyer M. (2015). VIH2 Regulates the Synthesis of Inositol Pyrophosphate InsP 8 and Jasmonate-Dependent Defenses in Arabidopsis. Plant. Cell.

[76] Phillippy B.O., Johnston M.R. (2006). Determination of Phytic Acid in Foods by Ion Chromatography with Post-Column Derivatization. J. Food Sci..

[77] Smith R., Martell A. (1976). Critical Stability Constants Volume 4: Inorganic Complexes.

[78] Fruhbeck G., Alonso R., Marzo F., Santidrian S. (1995). A Modified Method for the Indirect Quantitative Analysis of Phytate in Foodstuffs. Anal. Biochem..

[79] Vaintraub I.A., Lapteva N.A. (1988). Colorimetric determination of phytate in unpurified extracts of seeds and the products of their processing. Anal. Biochem..

[80] Costa-Bauza A., Grases F., Gomila I., Rodriguez A., Prieto R.M., Tur F. (2012). A simple and rapid colorimetric method for determination of phytate in urine. Urol. Res..

[81] Sandberg A.-S., Larsen T., Sandström B. (1993). High Dietary Calcium Level Decreases Colonic Phytate Degradation in Pigs Fed a Rapeseed Diet. J. Nutr..

[82] Thies W. (1991). Determination of the Phytic Acid and Sinapic Acid Esters in Seeds of Rapeseed and Selection of Genotypes with Reduced Concentrations of these Compounds. Fett Wiss. Technol. Sci. Technol..

[83] Cilliers J.J.L., Van Niekerk P.J. (1986). LC determination of phytic acid in food by postcolumn colorimetric detection. J. Agric. Food Chem..

[84] Rounds M.A., Nielsen S.S. (1993). Anion-exchange high-performance liquid chromatography with post-column detection for the analysis of phytic acid and other inositol phosphates. J. Chromatogr. A.

[85] Bos K.D., Verbeek C., Van Eeden C.H.P., Slump P., Wolters M.G.E. (1991). Improved determination of phytate by ion-exchange chromatography. J. Agric. Food Chem..

[86] Li N., Hefferren J.J., Li K. (2013). Quantitative Chemical Analysis.

[87] Meek J.L. (1986). Inositol bis-, tris-, and tetrakis(phosphate)s: Analysis in tissues by HPLC. Proc. Natl. Acad. Sci. USA.

[88] Mayr G.W. (1988). A novel metal-dye detection system permits picomolar-range h.p.l.c. analysis of inositol polyphosphates from non-radioactively labelled cell or tissue specimens. Biochem. J..

[89] Guse A.H., Emmrich F. (1991). T-cell receptor-mediated metabolism of inositol polyphosphates in Jurkat T-lymphocytes. Identification of a D-myo-inositol 1,2,3,4,6-pentakisphosphate-2-phosphomonoesterase activity, a D-myo-inositol 1,3,4,5,6-pentakisphosphate-1/3-phosphatase activity an. J. Biol. Chem..

[90] Mayr G.W., Thieleczek R. (1991). Masses of inositol phosphates in resting and tetanically stimulated vertebrate skeletal muscles. Biochem. J..

[91] Freund W.-D., Mayr G.W., Tietz C., Schultz J.E. (1992). Metabolism of inositol phosphates in the protozoan Paramecium. Characterization of a novel inositol-hexakisphosphate-dephosphorylating enzyme. Eur. J. Biochem..

[92] Schlemmer U., Jany K.-D., Berk A., Schulz E., Rechkemmer G. (2001). Degradation of phytate in the gut of pigs—Pathway of gastrointestinal inositol phosphate hydrolysis and enzymes involved. Arch. Tierernaehrung.

[93] Guse A.H., Goldwich A., Weber K., Mayr G.W. (1995). Non-radioactive, isomer-specific inositol phosphate mass determinations: High-performance liquid chromatography-micro-metal-dye detection strongly improves speed and sensitivity of analyses from cells and micro-enzyme assays. J. Chromatogr. B Biomed. Sci. Appl..

[94] Skoglund E., Carlsson N.G., Sandberg A.S. (1997). Determination of Isomers of Inositol Mono- to Hexaphosphates in Selected Foods and Intestinal Contents Using High-Performance Ion Chromatography. J. Agric. Food Chem..

[95] Phillippy B.Q., Bland J.M. (1988). Gradient ion chromatography of inositol phosphates. Anal. Biochem..

[96] Phillippy B.Q., Bland J.M., Evens T.J. (2003). Ion Chromatography of Phytate in Roots and Tubers. J. Agric. Food Chem..

[97] Skoglund E., Carlsson N.G., Sandberg A.S. (1998). High-Performance Chromatographic Separation of Inositol Phosphate Isomers on Strong Anion Exchange Columns. J. Agric. Food Chem..

[98] Smith R.E., MacQuarrie R.A. (1988). Determination of inositol phosphates and other biologically important anions by ion chromatography. Anal. Biochem..

[99] Smith R.E., MacQuarrie R.A., Jope R.S. (1989). Determination of Inositol Phosphates and Other Anions in Rat Brain. J. Chromatogr. Sci..

[100] Talamond P., Gallon G., Treche S. (1998). Rapid and sensitive liquid chromatographic method using a conductivity detector for the determination of phytic acid in food. J. Chromatogr. A.

[101] Skoglund E., Carlsson N.G., Sandberg A.S. (1997). Analysis of Inositol Mono- and Diphosphate Isomers Using High-Performance Ion Chromatography and Pulsed Amperometric Detection. J. Agric. Food Chem..

[102] Guse A.H., Emmrich F. (1992). Determination of inositol polyphosphates from human T-lymphocyte cell lines by anion-exchange high-performance liquid chromatography and post-column derivatization. J. Chromatogr. A.

[103] Hull S.R., Montgomery R. (1995). myo-Inositol Phosphates in Corn Steep Water. J. Agric. Food Chem..

[104] Chen Q.-C., Li B.W. (2003). Separation of phytic acid and other related inositol phosphates by high-performance ion chromatography and its applications. J. Chromatogr. A.

[105] Michalski R. (2018). Ion Chromatography Applications in Wastewater Analysis. Separations.

[106] Chen Q. (2004). Determination of Phytic Acid and Inositol Pentakisphosphates in Foods by High-Performance Ion Chromatography. J. Agric. Food Chem..

[107] Harland B.F., Smikle-Williams S., Oberleas D. (2004). High performance liquid chromatography analysis of phytate (IP6) in selected foods. J. Food Compos. Anal..

[108] Pourghasem G.B., Mahboub S.A., Razavieh S.V. (2005). Phytic acid and its molar ratio to zinc in consumed breads in tabriz. J. Urmia Univ. Med. Sci..

[109] Sekiguchi Y., Matsunaga A., Yamamoto A., Inoue Y. (2000). Analysis of condensed phosphates in food products by ion chromatography with an on-line hydroxide eluent generator. J. Chromatogr. A.

[110] Shintani H., Dasgupta P.K. (1987). Gradient anion chromatography with hydroxide and carbonate eluents using simultaneous conductivity and pH detection. Anal. Chem..

[111] Matsunaga A., Yamamoto A., Kurokawa H., Sekiguchi Y. (1998). Determination of condensed phosphates in food stuffs by ion chromatography. J. Food Hyg. Soc. Japan.

[112] Strong D.L., Dasgupta P.K., Friedman K., Stillian J.R. (1991). Electrodialytic Eluent Production and Gradient Generation in Ion Chromatography. Anal. Chem..

[113] Blaabjerg K., Hansen-Møller J., Poulsen H.D. (2010). High-performance ion chromatography method for separation and quantification of inositol phosphates in diets and digesta. J. Chromatogr. B.

[114] Blaabjerg K., Poulsen H.D. (2010). Microbial phytase and liquid feeding increase phytate degradation in the gastrointestinal tract of growing pigs. Livest. Sci..

[115] Liu X., Villalta P.W., Sturla S.J. (2009). Simultaneous determination of inositol and inositol phosphates in complex biological matrices: Quantitative ion-exchange chromatography/tandem mass spectrometry. Rapid Commun. Mass Spectrom..

[116] Kindt E., Shum Y., Badura L., Snyder P.J., Brant A., Fountain S., Szekely-Klepser G. (2004). Development and validation of an LC/MS/MS procedure for the quantification of endogenous myo-inositol concentrations in rat brain tissue homogenates. Anal. Chem..

[117] Sun M., Jaisi D.P. (2018). Distribution of inositol phosphates in animal feed grains and excreta: Distinctions among isomers and phosphate oxygen isotope compositions. Plant. Soil.

[118] von Sperber C., Tamburini F., Brunner B., Bernasconi S.M., Frossard E. (2015). The oxygen isotope composition of phosphate released from phytic acid by the activity of wheat and Aspergillus niger phytase. Biogeosciences.

[119] Ito M., Fujii N., Wittwer C., Sasaki A., Tanaka M., Bittner T., Jessen H.J., Saiardi A., Takizawa S., Nagata E. (2018). Hydrophilic interaction liquid chromatography–tandem mass spectrometry for the quantitative analysis of mammalian-derived inositol poly/pyrophosphates. J. Chromatogr. A.

[120] Kennington A.S., Hill C.R., Craig J., Bogardus C., Raz I., Ortmeyer H.K., Hansen B.C., Romero G., Larner J. (1990). Low Urinary chiro -Inositol Excretion in Non-Insulin-Dependent Diabetes Mellitus. N. Engl. J. Med..

[121] Haga H., Nakajima T. (1989). Determination of polyol profiles in human urine by capillary gas chromatography. Biomed. Chromatogr..

[122] Jansen G., Muskiet F.A.J., Schierbeek H., Berger R., van der Slik W. (1986). Capillary gas chromatographic profiling of urinary, plasma and erythrocyte sugars and polyols as their trimethylsilyl derivatives, preceded by a simple and rapid prepurification method. Clin. Chim. Acta.

[123] de Koning A.J. (1994). Determination of myo-inositol and phytic acid by gas chromatography using scyllitol as internal standard. Analyst.

[124] Roberts R.N., Johnston J.A., Fuhr B.W. (1965). A method for the quantitative estimation of myoinositol by gas-liquid chromatography. Anal. Biochem..

[125] March J., Simonet B.., Grases F. (2001). Determination of phytic acid by gas chromatography–mass spectroscopy: Application to biological samples. J. Chromatogr. B Biomed. Sci. Appl..

[126] March J.G., Forteza R., Grases F. (1996). Determination of inositol isomers and arabitol in human urine by gas chromatography-mass spectrometry. Chromatographia.

[127] Park H.-R., Ahn H.-J., Kim S.-H., Lee C.-H., Byun M.-W., Lee G.-W. (2006). Determination of the phytic acid levels in infant foods using different analytical methods. Food Control..

[128] Harland B.F., Oberleas D., Ellis R., Gelroth J., Gordon D., Phillips K., Ranhotra G., Shah B.G., Stoecker B., Trick K.D. (1986). Anion-Exchange Method for Determination of Phytate in Foods: Collaborative Study. J. AOAC Int..

[129] Lehrfeld J., Morris E.R. (1992). Overestimation of phytic acid in foods by the AOAC anion-exchange method. J. Agric. Food Chem..

[130] Schormüller J., Würdig G. (1957). Über das Vorkommen von Phytin, insbesondere in Getreide und Getreideprodukten. Deut. Leb. Rundschau.

[131] Angyal S.J., Russell A.F. (1969). Cyclitols. XXVIII. Methyl esters of inositol phosphates. The structure of phytic acid. Aust. J. Chem..

[132] Emilsson A., Sundler R. (1984). Differential activation of phosphatidylinositol deacylation and a pathway via diphosphoinositide in macrophages responding to zymosan and ionophore A23187. J. Biol. Chem..

[133] Kates M. (1972). Techniques of Lipidology. Laboratory Techniques in Biochemistry and Molecular Biology.

[134] Hatzack F., Rasmussen S.K. (1999). High-performance thin-layer chromatography method for inositol phosphate analysis. J. Chromatogr. B Biomed. Sci. Appl..

[135] Bandurski R.S., Axelrod B. (1951). The chromatographic identification of some biologically important phosphate esters. J. Biol. Chem..

[136] Shi J., Wang H., Hazebroek J., Ertl D.S., Harp T. (2005). The maize low-phytic acid 3 encodes a myo-inositol kinase that plays a role in phytic acid biosynthesis in developing seeds. Plant. J..

[137] Arnold P.W. (1956). Paper ionophoresis of inositol phosphates, with a note on the acid hydrolysates of phytic acid. Biochim. Biophys. Acta.

[138] Seiffert U.B., Agranoff B.W. (1965). Isolation and separation of inositol phosphates from hydrolysates of rat tissues. Biochim. Biophys. Acta Lipids Lipid Metab..

[139] Tate M.E. (1968). Separation of myoinositol pentaphosphates by moving paper electrophoresis (MPE). Anal. Biochem..

[140] Jackson J.F., Jones G., Linskens H.F. (1982). Phytic acid in pollen. Phytochemistry.

[141] Kikunaga S., Takahashi M., Huzisige H. (1985). Accurate and simple measurement of phytic acid contents in cereal grains. Plant. Cell Physiol..

[142] Blatny P., Kvasnicka F., Kenndler E. (1995). Determination of Phytic Acid in Cereal Grains, Legumes, and Feeds by Capillary Isotachophoresis. J. Agric. Food Chem..

[143] Nardi A., Cristalli M., Desiderio C., Ossicini L., Shukla S.K., Fanali S. (1992). Indirect UV photometric detection in capillary zone electrophoresis for the determination of phytate in soybeans. J. Microcolumn Sep..

[144] Kvasnička F., Ševčík R., Voldřich M., Krátká J. (2004). Determination of aristolochic acid by capillary zone electrophoresis. Open Chem..

[145] Kvasnička F., Čopíková J., Ševčík R., Václavíková E., Synytsya A., Vaculová K., Voldřich M. (2011). Determination of phytic acid and inositolphosphates in barley. Electrophoresis.

[146] Losito O., Szijgyarto Z., Resnick A.C., Saiardi A. (2009). Inositol pyrophosphates and their unique metabolic complexity: Analysis by gel electrophoresis. PLoS ONE.

[147] Alimohammadi M., Ali N., Khodakovskaya M. (2013). Quantitative Detection of Inositol Hexakisphosphate (InsP6) in Crop Plants Using Polyacrylamide Gel Electrophoresis (PAGE). Am. J. Plant. Sci..

[148] Giridhari A.C., Sujatha R., Deeshma K.P. (2017). Detection and quantification of phytic acid in black pepper variety panniyur-1 using polyacrylamide gel electrophoresis. J. Trop. Agric..

[149] Wilson M.S.C., Bulley S.J., Pisani F., Irvine R.F., Saiardi A. (2015). A novel method for the purification of inositol phosphates from biological samples reveals that no phytate is present in human plasma or urine. Open Biol..

[150] Wilson M., Saiardi A. (2018). Inositol Phosphates Purification Using Titanium Dioxide Beads. Bio Protoc..

[151] Qiu D., Wilson M.S., Eisenbeis V.B., Harmel R.K., Riemer E., Haas T.M., Wittwer C., Jork N., Gu C., Shears S.B. (2020). Analysis of inositol phosphate metabolism by capillary electrophoresis electrospray ionization mass spectrometry. Nat. Commun..

[152] Pavlovic I., Thakor D.T., Vargas J.R., McKinlay C.J., Hauke S., Anstaett P., Camunã R.C., Bigler L., Gasser G., Schultz C. (2016). Cellular delivery and photochemical release of a caged inositol-pyrophosphate induces PH-domain translocation in cellulo. Nat. Commun..

[153] Harmel R.K., Puschmann R., Nguyen Trung M., Saiardi A., Schmieder P., Fiedler D. (2019). Harnessing 13 C-labeled myo -inositol to interrogate inositol phosphate messengers by NMR. Chem. Sci..

[154] Lolas G.M., Palamidis N., Markakis P. (1976). Phytic acid total phosphorus relationship relationship in barley, oats, soybeans and wheat. Cereal Chem..

[155] March J.G., Villacampa A.I., Grases F. (1995). Enzymatic—spectrophotometric determination of phytic acid with phytase from Aspergillus ficuum. Anal. Chim. Acta.

[156] Raheja R.K., Kaur C., Singh A., Bhatia I.S. (1973). New calorimetric method for the quantitative estimation of phospholipids without acid digestion. J. Lipid Res..

[157] Mohamed A.I., Perera P.A.J., Hafez Y.S. (1986). New Chromophore for Phytic Acid Determination. Cereal Chem..

[158] Santiviago C., Peralta J., López I. (2020). A simple spectrophotometric method to determine phytic acid in poultry wastewater without acid digestion. Int. J. Environ. Anal. Chem..

[159] Carneiro J.M.T., Zagatto E.A.G., Santos J.L.M., Lima J.L.F.C. (2002). Spectrophotometric determination of phytic acid in plant extracts using a multi-pumping flow system. Anal. Chim. Acta.

[160] Agostinho A.J., de Souza Oliveira W., Anunciação D.S., Santos J.C.C. (2016). Simple and Sensitive Spectrophotometric Method for Phytic Acid Determination in Grains. Food Anal. Methods.

[161] Lee M.H. (1995). Official Methods of Analysis of AOAC International.

[162] Frølich W., Drakenberg T., Asp N.-G. (1986). Enzymic degradation of phytate (myo-inositol Hexaphosphate) in whole grain flour suspension and dough. A comparison between 31P NMR spectroscopy and a ferric ion method. J. Cereal Sci..

[163] McKie V.A., McCleary B. (2016). V A Novel and Rapid Colorimetric Method for Measuring Total Phosphorus and Phytic Acid in Foods and Animal Feeds. J. AOAC Int..

[164] Perera I., Fukushima A., Arai M., Yamada K., Nagasaka S., Seneweera S., Hirotsu N. (2019). Identification of low phytic acid and high Zn bioavailable rice (Oryza sativa L.) from 69 accessions of the world rice core collection. J. Cereal Sci..

[165] Kamaya M., Furuki T., Nagashima K., Ishii E., Saito H. (1995). Indirect spectrophotometric determination of phytic acid with zinc chloranilate. Phytochem. Anal..

[166] March J.G., Simonet B.M., Grases F. (1999). Fluorimetric determination of phytic acid based on the activation of the oxidation of 2,2′-dipyridyl ketone hydrazone catalysed by Cu(II). Analyst.

[167] Chen Y., Chen J., Ma K., Cao S., Chen X. (2007). Fluorimetric determination of phytic acid in urine based on replacement reaction. Anal. Chim. Acta.

[168] Cao S., Dong N., Chen J. (2011). Synchronous fluorescence determination of phytic acid in FOODSTUFFS and urine based on replacement reaction. Phytochem. Anal..

[169] Kolozsvari B., Parisi F., Saiardi A. (2014). Inositol phosphates induce DAPI fluorescence shift. Biochem. J..

[170] Kolozsvari B., Firth S., Saiardi A. (2015). Raman Spectroscopy Detection of Phytic Acid in Plant Seeds Reveals the Absence of Inorganic Polyphosphate. Mol. Plant..

[171] Chen Y., Chen J., Luo Z., Ma K., Chen X. (2009). Synchronous fluorescence analysis of phytate in food. Microchim. Acta.

[172] Qu Z., Na W., Nie Y., Su X. (2018). A novel fluorimetric sensing strategy for highly sensitive detection of phytic acid and hydrogen peroxide. Anal. Chim. Acta.

[173] Bebot-Brigaud A., Dange C., Fauconnier N., Gérard C. (1999). ^31^P NMR, potentiometric and spectrophotometric studies of phytic acid ionization and complexation properties toward Co^2+^, Ni^2+^, Cu^2+^, Zn^2+^ and Cd^2+^. J. Inorg. Biochem..

[174] Šala M., Makuc D., Kolar J., Plavec J., Pihlar B. (2011). Potentiometric and 31P NMR studies on inositol phosphates and their interaction with iron(III) ions. Carbohydr. Res..

[175] Tse R.S., Wong S.C., Yuen C.P. (1980). Determination of deuterium/hydrogen ratios in natural waters by Fourier transform nuclear magnetic resonance spectrometry. Anal. Chem..

[176] Harland B.F., Oberleas D. (1977). A modified method for phytate analysis using an ion-exchange procedure: Application to textured vegetable proteins [Soybeans]. Cereal Chem..

[177] Mazzola E.P., Phillippy B.Q., Harland B.F., Miller T.H., Potemra J.M., Katsimpiris E.W. (1986). Phosphorus-31 nuclear magnetic resonance spectroscopic determination of phytate in foods. J. Agric. Food Chem..

[178] Johnson K., Barrientos L.G., Le L., Murthy P.P.N. (1995). Application of Two-Dimensional Total Correlation Spectroscopy for Structure Determination of Individual Inositol Phosphates in a Mixture. Anal. Biochem..

[179] Turner B.L. (2007). Inositol phosphates in soil: Amounts, forms and significance of the phosphorylated inositol stereoisomers. Inositol Phosphates: Linking Agriculture and the Environment.

[180] Doolette A.L., Smernik R.J., Dougherty W.J. (2011). Overestimation of the importance of phytate in NaOH–EDTA soil extracts as assessed by 31P NMR analyses. Org. Geochem..

[181] Wu J., Paudel P., Sun M., Joshi S.R., Stout L.M., Greiner R., Jaisi D.P. (2015). Mechanisms and Pathways of Phytate Degradation: Evidence from Oxygen Isotope Ratios of Phosphate, HPLC, and Phosphorus-31 NMR Spectroscopy. Soil Sci. Soc. Am. J..

[182] Watson F.T., Smernik R.J., Doolette A.L. (2019). Thermal degradation of phytate produces all four possible inositol pentakisphosphates as determined by ion chromatography and 1 H and 31 P NMR spectroscopy. Phosphorus. Sulfur. Silicon Relat. Elem..

[183] Plaami S., Kumpulainen J. (1991). Determination of Phytic Acid in Cereals Using ICP-AES to Determine Phosphorus. J. AOAC Int..

[184] Saastamoinen M., Plaami S., Kumpulainen J. (1992). β-Glucan and Phytic Acid Content of Oats Cultivated in Finland. Acta Agric. Scand. Sect. B Soil Plant. Sci..

[185] Grases F., Llobera A. (1996). Determination of Phytic Acid in Urine by ICP Atomic Emission Spectrometry. Anal. Lett..

[186] Grases F., Perelló J., Isern B., Prieto R.M. (2004). Determination of myo-inositol hexakisphosphate (phytate) in urine by inductively coupled plasma atomic emission spectrometry. Anal. Chim. Acta.

[187] Muñoz J.A., Valiente M. (2003). Determination of Phytic Acid in Urine by Inductively Coupled Plasma Mass Spectrometry. Anal. Chem..

[188] Muñoz J.A., López-Mesas M., Valiente M. (2010). Minimum handling method for the analysis of phosphorous inhibitors of urolithiasis (pyrophosphate and phytic acid) in urine by SPE-ICP techniques. Anal. Chim. Acta.

[189] Liu T., He L., Valiente M., López-Mesas M. (2017). Fast determination of bioactive phytic acid and pyrophosphate in walnuts using microwave accelerated extraction. Food Chem..

[190] Mak W.C., Ng Y.M., Chan C., Kwong W.K., Renneberg R. (2004). Novel biosensors for quantitative phytic acid and phytase measurement. Biosens. Bioelectron..

[191] Caseli L., Moraes M.L., Zucolotto V., Ferreira M., Nobre T.M., Zaniquelli M.E.D., Rodrigues Filho U.P., Oliveira O.N. (2006). Fabrication of Phytic Acid Sensor Based on Mixed Phytase−Lipid Langmuir−Blodgett Films. Langmuir.

[192] Troitsky V.I., Berzina T.S., Pastorino L., Bernasconi E., Nicolini C. (2003). A new approach to the deposition of nanostructured biocatalytic films. Nanotechnology.

[193] Moraes M.L., Oliveira O.N., Filho U.P.R., Ferreira M. (2008). Phytase immobilization on modified electrodes for amperometric biosensing. Sens. Actuators B Chem..

[194] Moraes M.L., Maki R.M., Paulovich F.V., Rodrigues Filho U.P., de Oliveira M.C.F., Riul A., de Souza N.C., Ferreira M., Gomes H.L., Oliveira O.N. (2010). Strategies to Optimize Biosensors Based on Impedance Spectroscopy to Detect Phytic Acid Using Layer-by-Layer Films. Anal. Chem..

[195] Esmaeili C., Norouzi P., Zar M.S., Eskandari M., Faridbod F., Ganjali M.R. (2019). A FFT Square Wave Voltammetry Sensing Method for Highly Sensitive Detection of Phytic Acid Using a Cerium Oxide Nanoparticles Decorated Graphene Oxide. J. Electrochem. Soc..

[196] Shi H., Zhang A., Du H., Zhang M., Zhang Y., Huang H., Xiao Y., Zhang Y., He X., Wang K. (2019). A novel fluorescent nanosensor based on small-sized conjugated polyelectrolyte dots for ultrasensitive detection of phytic acid. Talanta.

[197] Lee M., Moon J.H., Jun E.J., Kim G., Kwon Y.-U., Lee J.Y., Yoon J. (2014). A tetranaphthoimidazolium receptor as a fluorescent chemosensor for phytate. Chem. Commun..

[198] Wang Y., Ma X., Wen Y., Zheng Y., Duan G., Zhang Z., Yang H. (2010). Phytic Acid-Based Layer-by-Layer Assembly for Fabrication of Mesoporous Gold Film and Its Biosensor Application. J. Electrochem. Soc..

[199] Dai H., Wang N., Wang D., Ma H., Lin M. (2016). An electrochemical sensor based on phytic acid functionalized polypyrrole/graphene oxide nanocomposites for simultaneous determination of Cd(II) and Pb(II). Chem. Eng. J..

[200] Wang K., Liu P., Ye Y., Li J., Zhao W., Huang X. (2014). Fabrication of a novel laccase biosensor based on silica nanoparticles modified with phytic acid for sensitive detection of dopamine. Sens. Actuators B Chem..

[201] Yang L., Wang H., Lü H., Hui N. (2020). Phytic acid doped poly(3,4-ethylenedioxythiophene) modified with copper nanoparticles for enzymeless amperometric sensing of glucose. Microchim. Acta.

